# Brief Report: Inhibition of miR‐145 Enhances Reprogramming of Human Dermal Fibroblasts to Induced Pluripotent Stem Cells

**DOI:** 10.1002/stem.2220

**Published:** 2015-10-09

**Authors:** Tomas Barta, Lucie Peskova, Joseph Collin, David Montaner, Irina Neganova, Lyle Armstrong, Majlinda Lako

**Affiliations:** ^1^ International Clinical Research CenterSt. Anne's University Hospital BrnoBrnoCzech Republic; ^2^ Department of Histology and Embryology, Faculty of MedicineMasaryk UniversityBrnoCzech Republic; ^3^ Institute of Genetic MedicineNewcastle University, International Centre for Life, Newcastle upon TyneUnited Kingdom; ^4^ Centro de Investigación Príncipe FelipeValenciaSpain

**Keywords:** microRNA, miR‐145, Induced pluripotent stem cells, Reprogramming, Mesenchymal‐to‐epithelial transition, OCT4, SOX2, KLF4, c‐MYC

## Abstract

MicroRNA (miRNAs) are short noncoding RNA molecules involved in many cellular processes and shown to play a key role in somatic cell induced reprogramming. We performed an array based screening to identify candidates that are differentially expressed between dermal skin fibroblasts (DFs) and induced pluripotent stem cells (iPSCs). We focused our investigations on miR‐145 and showed that this candidate is highly expressed in DFs relative to iPSCs and significantly downregulated during reprogramming process. Inhibition of miR‐145 in DFs led to the induction of “cellular plasticity” demonstrated by: (a) alteration of cell morphology associated with downregulation of mesenchymal and upregulation of epithelial markers; (b) upregulation of pluripotency‐associated genes including SOX2, KLF4, C‐MYC; (c) downregulation of miRNA let‐7b known to inhibit reprogramming; and (iv) increased efficiency of reprogramming to iPSCs in the presence of reprogramming factors. Together, our results indicate a direct functional link between miR‐145 and molecular pathways underlying reprogramming of somatic cells to iPSCs. Stem Cells *2016;34:246–251*

**Significance Statement**

iPSCs can be generated directly and conveniently from healthy and affected individuals and have very significant potential for regenerative medicine, disease modeling, basic biology, and drug discovery studies. However, there are limitations in the technology and the induction process, which is currently not well‐understood. Recent studies indicate that short noncoding RNAs, named miRNAs play an important role in various stages of somatic cell reprogramming to iPSC. In this study, we performed an expression based analysis to identify candidate miRNAs that are highly expressed in somatic cells but are downregulated during the reprogramming process. This led us to identification of miR‐145 as a key miRNA candidate involved in the reprogramming process. In this manuscript, we show that miR‐145 is highly expressed in human dermal fibroblasts but significantly downregulated during the reprogramming process to iPSC. Our data suggests that inhibition of miR‐145 leads to induction of cellular plasticity as demonstrated by mesenchymal to epithelial transition, upregulation of pluripotency markers, and increased efficiency of reprogramming.

## Introduction

Somatic cell induced reprogramming requires comprehensive reconfiguration of cellular signalling pathways and molecular profiles, including microRNAs (miRNAs). miRNAs are short, noncoding RNA molecules (∼22 nucleotides in length) that play crucial active role in many cellular processes by post‐transcriptional regulation of specific messenger RNA (mRNA). Although some miRNAs have been shown to play an important role during various stages of reprogramming 1, 2, 3, our detailed understanding of how miRNAs regulate this process remains elusive.

A number of studies have indicated that pluripotent stem cells [encompassing human embryonic stem cells (hESCs) and human induced pluripotent stem cells (iPSCs)] express a distinct set of miRNAs that underline the maintenance of the pluripotent phenotype by influencing various processes such as cell cycle, epigenetic profile, and resistance to apoptosis 3, 4, 5. It is not surprising that some of those pluripotency‐associated miRNAs also play an important role in cellular reprogramming mainly through regulation of mesenchymal to epithelial transition (MET), bypassing senescence or regulating the expression of key pluripotency factors. Among the later class, miR‐145 has recently been shown to inhibit stem cell properties and to regulate the expression of OCT4, SOX2, KLF4, and c‐MYC (further abbreviated as OKSM) in cancer and stem cells 6, 7, 8, 9, 10, 11, 12. However, the role of miR‐145 in reprogramming of somatic cells to iPSCs has not been elucidated.

Given the critical importance of miR‐145 on the regulation of OKSM factors and stem cell properties, we sought to study in detail the role of miR‐145 in reprogramming of human dermal skin fibroblasts (DFs) to iPSCs. Our array based expression analysis and reverse transcriptase quantitative polymerase chain reaction (qPCR) indicated that miR‐145 is highly expressed in DFs when compared to iPSCs and significantly downregulated during the reprogramming process. Furthermore, inhibition of miR‐145 in DFs resulted in a change of morphology from a long‐spindled fibroblast to short‐spindled cell shape associated with change of mesenchymal/epithelial genes toward epithelial phenotype, upregulation of SOX2, c‐MYC, KLF4, downregulation of miRNA let‐7b, and enhanced the reprogramming efficiency, suggesting a key role for this miRNA in somatic cell induced reprogramming.

## Materials and Methods

See Supporting Information Materials.

## Results and Discussion

To get insights into the expression profile of miRNAs involved in reprogramming process and to identify differentially expressed miRNAs, we performed miRNA expression analysis between DFs and iPSCs using Agilent human miRNA microarray. 38 miRNAs were significantly upregulated in iPSCs compared to DFs (Supporting Information Table 1) and 51 miRNAs were significantly upregulated in DFs compared to iPSCs (Supporting Information Table 2). The criteria for significant upregulation/downregulation were more than threefold change of expression and *p* value (<.05).

Inspection of predicted target genes suggests that these 89 miRNAs may be central to reprogramming. Likely target genes (Supporting Information Tables 1 and 2) include important histone deacetylases/methyltransferases, transcriptional repressors, chromatin modifiers, polycomb repressive complex members, DNA methylases, cell cycle regulators, p53 processing proteins, and so on. The impact of DNA methylase and histone deacetylase inhibition on reprogramming underlines the potential importance of these 89 miRNAs in controlling epigenetic regulation and hence the reprogramming process. Of the 89 candidates, we applied two selection criteria for identification of study candidates: (a) highly expressed in DFs relative to iPSCs and (b) miRNAs shown to regulate pluripotency factors. This led to selection of miR‐145 as a key candidate for this study.

**Figure 1:**
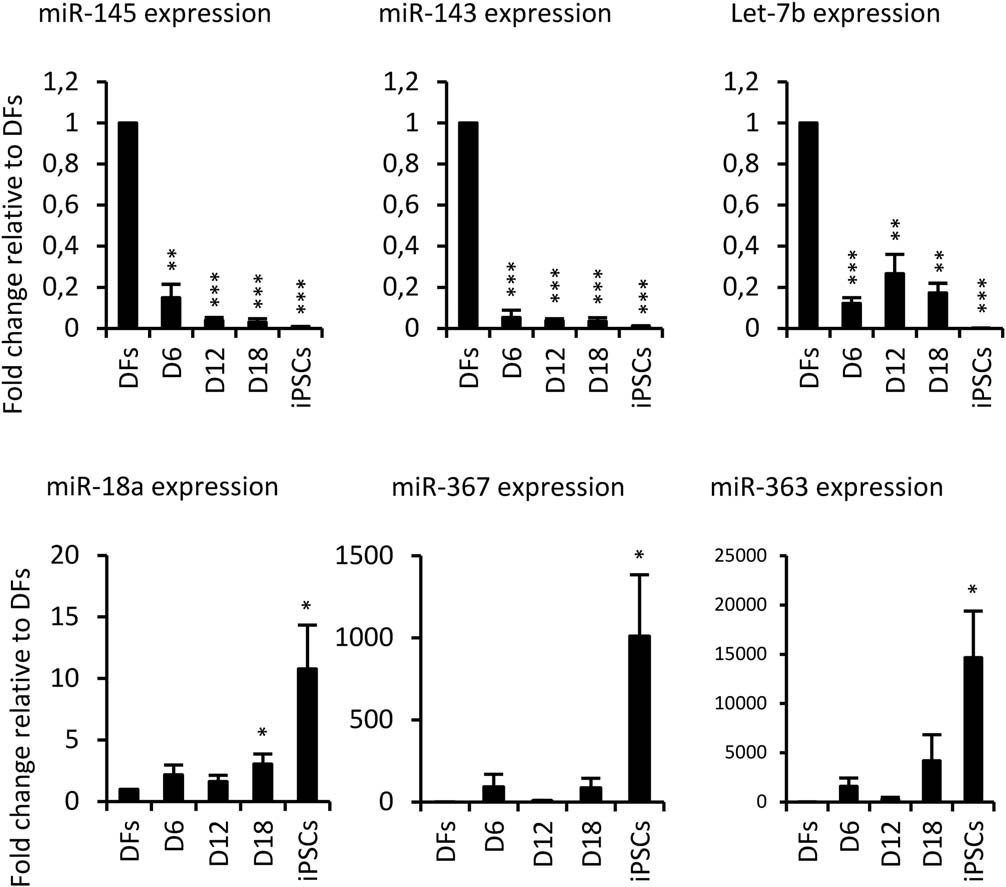
Expression of miR‐145, miR‐143, Let‐7b, miR‐18a, miR‐367, and miR‐363 at specific stages of reprogramming of DFs to iPSCs, as determined by reverse transcriptase quantitative polymerase chain reaction. Error bars show SD of three independent experiments using three different cell lines. Stars show statistical significance of miR‐145 expression between DFs and cells at specific stage of reprogramming (*, *p* < .05; **, *p* < .01; ***, *p* < .001). Abbreviations: DFs, dermal skin fibroblasts; iPSCs, induced pluripotent stem cells.

In order to assess the expression of miR‐145 at specific stages of reprogramming and confirm the array results, we transduced DFs with OKSM using CytoTune kit, harvested cells every 6 days, and assessed the expression of selected miRNAs (miR‐145, miR‐143, Let‐7b, miR‐18a, miR‐367, and miR‐363). As shown on Figure 1, the expression of miR‐145 was significantly downregulated as early as day 6 post‐transduction, thus corroborating previously published data in pluripotent stem cells 4, 5, 11. Furthermore, all the other qPCR tested miRNAs displayed the same expression pattern change as revealed by the microarray study.

**Figure 2:**
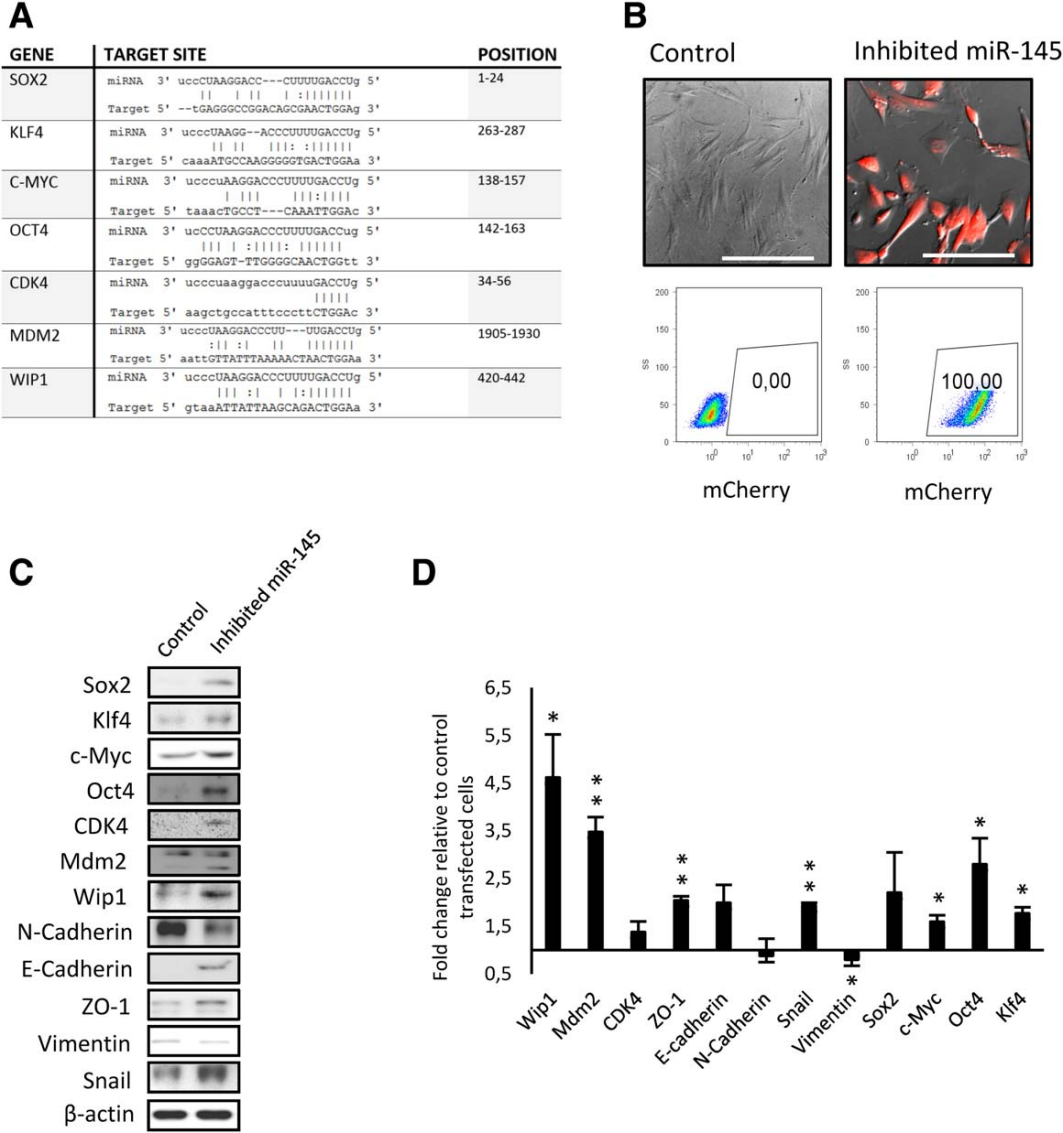
miR‐145 target validation. **(A)**: Summary of miR‐145 targets including target sites predicted by miRTarBase. **(B)**: DFs stably expressing miR‐145 inhibitor including mCherry reporter. The expression of mCherry reporter was determined using fluorescence microscopy and flow cytometry. Scale bar = 100 μm. **(C)**: Western blot analysis of miR‐145 targets and mesenchymal/epithelial markers in dermal skin fibroblasts (DFs) cell lines stably expressing miR‐145 inhibitor. β‐Actin was used as a loading control. This is a representative example of at least three independent biological replicates in the three DFs used in this study. **(D)**: Optical density of bands generated by Western blot analysis shown as fold change in expression in miR‐145 inhibited versus control transfected samples (*n* = 3). Stars show statistical significance (*, *p* < .05; **, *p* < .01).

Since miR‐145 is highly expressed in DFs and it is rapidly downregulated upon reprogramming to iPSCs, we hypothesized that its downregulation may lead to improved reprogramming. Using lentiviral particles containing miR‐145 inhibitor and mCherry reporter, we generated DFs cell lines stably inhibiting miR‐145. In order to test, if miR‐145 was inhibited, we scanned miRTarBase (version 4.5), a database of experimentally validated miRNA targets (http://mirtarbase.mbc.nctu.edu.tw/), for genes that are regulated by miR‐145. We selected seven target genes (Fig. 2A) and tested their expression upon miR‐145 inhibition at protein level using Western blot analysis. As shown on Figures 2C, 2D, we detected upregulation of SOX2, KLF4, c‐MYC, OCT4, CDK4, MDM2, and WIP1 indicating that miR‐145 was inhibited.

Notably, upon the inhibition of miR‐145 cells changed their shape from long‐spindled fibroblast shape to a short‐spindle epithelial morphology (Fig. 2B), suggesting a possible mesenchymal to epithelial transition. In order to test, whether miR‐145 inhibition affects the expression of mesenchymal/epithelial markers, we performed Western blot analysis of target proteins involved in MET process in DFs. As shown on Figures 2C, 2D, inhibition of miR‐145 upregulated the expression of E‐cadherin, ZO‐1, and reduced the expression of N‐cadherin and Vimentin, indicating downregulation of key mesenchymal and upregulation of epithelial markers. Wound healing and migration assays also indicated a slower migration capacity of cells transfected with miR‐145, providing further proof on the role of this miRNA in MET transition (Supporting Information Fig. S1). Contrary to these findings, mesenchymal marker Snail was significantly upregulated, suggesting that miR‐145 inhibition was not sufficient to fully promote/induce MET. Furthermore, Snail has been shown to be expressed during the early stages of reprogramming and to be a strong enhancer of reprogramming acting via the downregulation of miRNA let‐7 family 13, 14, and therefore upregulation of Snail expression may be more related to enhancement of iPSC generation rather than MET transition.

**Figure 3:**
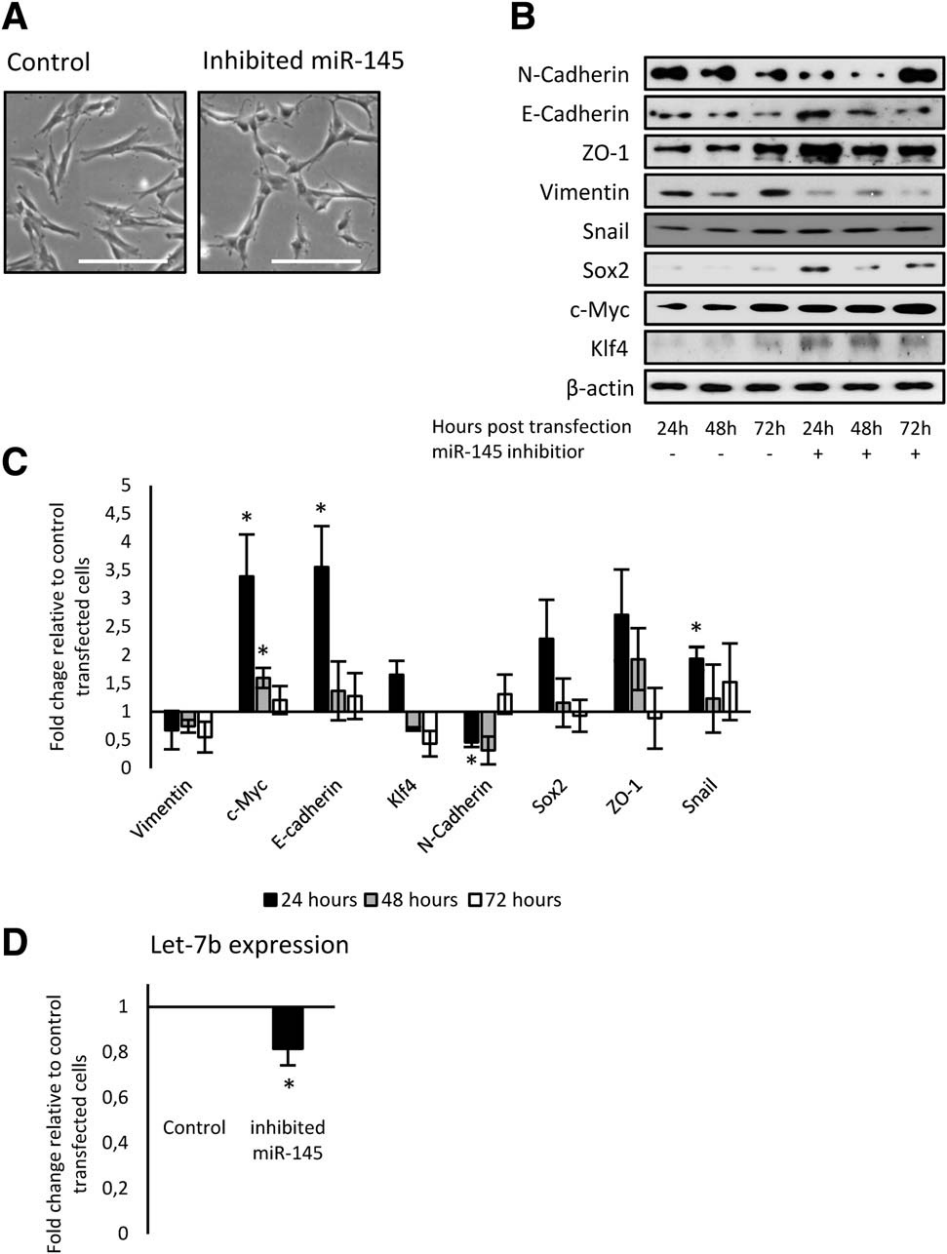
Characterization of DFs transiently inhibiting miR‐145. **(A)**: Morphology of human DFs transiently transfected by synthetic miR‐145 inhibitor 48 hours after transfection, as determined by light microscopy. Scale bar = 100 μm. **(B)**: Western blot analysis of miR‐145 targets and mesenchymal/epithelial markers in DFs cell lines transiently inhibiting miR‐145. β‐Actin was used as a loading control. This is a representative example of at least three independent biological replicates in the three DFs used in this study. **(C)**: Optical density of bands generated by Western blot analysis (*n* = 3). **(D)**: Let‐7b expression upon transient inhibition (24 hours) of miR‐145 in DFs, as determined by reverse transcriptase quantitative polymerase chain reaction. Error bars show SD of four independent experiments across three independent DFs used in this study. Stars show statistical significance (*, *p* < .05).

Given the importance of MET in the first steps of reprogramming process 15, 16, 17, 18, changes in expression of mesenchymal/epithelial markers and pluripotency markers upon miR‐145 inhibition in DFs (Fig. 2B–2D), we speculated that the efficiency of iPSCs generation could be enhanced by the inhibition of miR‐145 in DFs. Since stable inhibition of miR‐145 does not allow to inhibit miR‐145 at specific stage, and DFs stably expressing miR‐145 inhibitor undergo senescence after approximately two to three passages, presumably by intensive selection for hygromycin resistance (data not shown), we transfected DFs with a synthetic miR‐145 inhibitor which led to significant downregulation of miR‐145 (Supporting Information Fig. S2). Transient inhibition of miR‐145 in DFs led to the induction of similar morphology as in case of stabile inhibition (Fig. 3A). Furthermore, it led to downregulation of mesenchymal markers, upregulation of epithelial markers, and upregulation of SOX2, KLF4, and c‐MYC (Fig. 3B, 3C; Supporting Information Fig. S3). For the majority of the markers tested, these impacts were more pronounced at 24–48 hours post‐miR‐145 inhibition very likely due to transient nature of miR‐145 inhibition used for this assay. Notably, upregulation of Snail was observed upon both stable and transient inhibition of miR‐145 (Figs. 2C, 2D, 3B, 3C). In order to test, if inhibition of miR‐145 downregulates let‐7 via upregulation of Snail, we compared the expression of let‐7b in nontransfected DFs versus DFs with transiently inhibited miR‐145. We found that let‐7b was downregulated by approximately 20% upon inhibition of miR‐145 (Fig. 3D), indicating a possible link between miR‐145 and let‐7 during reprogramming.

**Figure 4:**
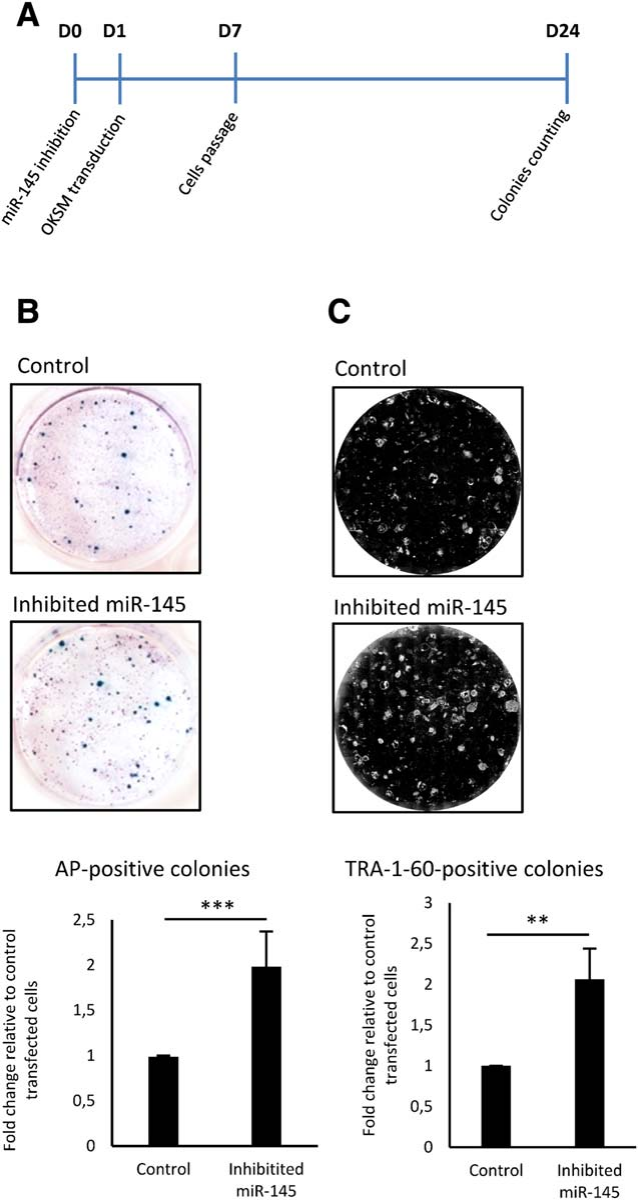
Efficiency of induced pluripotent stem cells generation upon inhibition of miR‐145. **(A)**: Schematic presentation of experimental work flow. **(B)**: Assessment of the number of colonies positive by AP activity. Error bars show SD of seven experiments using three different cell lines. **(C)**: Assessment of the number of colonies positive for TRA‐1‐60 expression, as determined by indirect immunofluorescence. Error bars show SD of five experiments using three different cell lines. Stars show statistical significance (**, *p* < .01; ***, *p* < .001).

To assess how inhibition of miR‐145 affects iPSCs generation efficiency, we transiently transfected DFs with miR‐145 inhibitor at day 0. The next day cells were transduced with OKSM and 23 days later the efficiency of iPSCs generation was determined by counting of AP‐positive and TRA‐1‐60‐positive colonies (Fig. 4A for a representative summary). The inhibition of miR‐145 at day 0 increased the reprogramming efficiency by approximately twofold (Fig. 4B, 4C); however inhibitor at later stages (day 7) did not significantly increased iPSCs generation (data not shown).

miR‐143 and miR‐145 belong to the same miRNA cluster and is possible that there may exist functional redundancy between these two miRNAs. To investigate this further, we performed single miR‐143 inhibition and combined miR‐143 and miR‐145 inhibitions, but we did not see a significant increase in reprogramming efficiency in either case (data not shown). Furthermore, miRTarBase analysis indicated different subsets of target genes for each of these two miRNAs, suggesting lack of functional redundancy.

## Summary

Taken together, out data show that the expression of miR‐145 is rapidly downregulated during reprogramming of somatic cells into iPSCs. Furthermore, inhibition of miR‐145 in DFs downregulates mesenchymal markers, upregulates epithelial markers, upregulates SOX2, KLF4, and c‐MYC expression, and downregulates let‐7b, thus facilitating and enhancing reprogramming of DFs to iPSCs.

## Author Contributions

T.B.: conception and design, collection and/or assembly of data, fund raising, data analysis and interpretation, manuscript writing, and final approval of manuscript; L.P., J.C., and D.M.: collection and/or assembly of data, data analysis and interpretation, and final approval of manuscript; I.N.: data analysis and interpretation and final approval of manuscript; L.A.: conception and design, data analysis and interpretation, fund raising, and final approval of manuscript; M.L.: conception and design, data analysis and interpretation, manuscript writing, fund raising, and final approval of manuscript.

## Disclosure of Potential Conflict of Interest

The authors indicate no potential conflict of interest.

## Supporting information

Additional Supporting Information may be found in the online version of this article

Supporting Information

Supporting Information

Supporting Information

Supporting Information

Supporting Information

Supporting Information
