# Supplementary material for: Brief Report: Inhibition of miR‐145 Enhances Reprogramming of Human Dermal Fibroblasts to Induced Pluripotent Stem Cells
Source: Stem Cells. 2015 Oct 9;34(1):246–51. doi: 10.1002/stem.2220 (PMC4982107; doi:10.1002/stem.2220)
Supplement: Supplementary file 3 — Supporting Information [file STEM-34-246-s003.docx]

**Supplemental Table 1**

| **miRNA species** | **Fold change [log] upregulation in human dermal fibroblasts versus iPSCs** | **Adjusted p-value** | **Predicted target gene involved in cell cycle regulation and DNA repair** | **Predicted target gene involved in chromatin modelling and transcription** | **Predicted target gene involved in cell proliferation or apoptosis** | **Predicted target gene involved in maintenance of pluripotency** | **Predicted target gene involved in key signalling pathways** |
| --- | --- | --- | --- | --- | --- | --- | --- |
| **hsa-miR-367** | 10.64 | 6.465E-217 | *p57, p27,* | - | - | **Unique expression in hESCs** | *-* |
| **hsa-miR-302a** | 9.23 | 1.155E-71 | *CDK2,*  *CDK1, NEK9* | *ARID4B,*  *SIN3A, SUV39H, MECP2, HDAC4* | - | **Unique expression in hESCs** | *LEFTY 1, DKK1* |
| **hsa-miR-302c** | 7.88 | 2.437E-58 | *CDK2,*  *CDK1, NEK9* | *ARID4B,*  *SIN3A, SUV39H, MECP2, HDAC4* | - | **Unique expression in hESCs** | *LEFTY 1, DKK1* |
| **hsa-miR-302a*** | 7.65 | 2.155E-30 | *-* | *-* | *MORF4L* | **Unique expression in hESCs** | *GSK3B* |
| **hsa-miR-302d** | 6.10 | 7.325E-07 | *CDK2,*  *CDK1, NEK9* | *ARID4B,*  *SIN3A, SUV39H, MECP2, HDAC4* | *-* | **Unique expression in hESCs** | *LEFTY 1, DKK1* |
| hsa-miR-96 | 5.62 | 1.208E-85 | *CHES1, CDC37, PAK1, CDK5R2* | *JMJD1C,*  *NIPBL, LMNA* | *MORF4L1,* | *LMNA, ACVR1* | *-* |
| **hsa-miR-302b** | 5.59 | 3.965E-10 | *CDK2,*  *CDK1, NEK9* | *ARID4B,SIN3A, SUV39H, MECP2, HDAC4* | *LATS2,tp63* | **Unique expression in hESCs** | *LEFTY 1, DKK1* |
| hsa-miR-18a | 5.26 | 8.837E-59 | *ATM, CDK2, CDC2L6, CDC42, CCND2* | *-* | *-* | *DICER1, LIN28, SMAD2* | *-* |
| hsa-miR-18b | 4.73 | 1.920E-28 | *ATM, CCND2* | *JARID1* | *-* | *DICER1* | *-* |
| hsa-miR-197 | 4.48 | 3.219E-89 | *CCNL1* | *-* | *-* | - | *RAB28, SMG1, MAPK4, MAP2K1, KRAS2* |
| hsa-miR-886-3p | 4.29 | 3.498E-16 | *-* | *-* | *-* | - | *RALGPS1* |
| hsa-miR-205 | 4.20 | 3.343E-46 | *CDK11, RAD17* | *SBF2* | *-* | - | *PLCB1* |
| hsa-miR-20b | 4.11 | 1.679E-90 | [*CCNG2*](http://www.ncbi.nlm.nih.gov/sites/entrez?Db=gene&Cmd=ShowDetailView&TermToSearch=901)*,* [*CDC23*](http://www.ncbi.nlm.nih.gov/sites/entrez?Db=gene&Cmd=ShowDetailView&TermToSearch=8697)*,* [*CDKN1A*](http://www.ncbi.nlm.nih.gov/sites/entrez?Db=gene&Cmd=ShowDetailView&TermToSearch=1026)*,* [*PAK7*](http://www.ncbi.nlm.nih.gov/sites/entrez?Db=gene&Cmd=ShowDetailView&TermToSearch=57144)*,* [*CCND1*](http://www.ncbi.nlm.nih.gov/sites/entrez?Db=gene&Cmd=ShowDetailView&TermToSearch=595)*,* [*CCND2*](http://www.ncbi.nlm.nih.gov/sites/entrez?Db=gene&Cmd=ShowDetailView&TermToSearch=894) | [*ARID4A*](http://www.ncbi.nlm.nih.gov/sites/entrez?Db=gene&Cmd=ShowDetailView&TermToSearch=5926) | [*PDCD1LG2*](http://www.ncbi.nlm.nih.gov/sites/entrez?Db=gene&Cmd=ShowDetailView&TermToSearch=80380)*,* [*CASP2*](http://www.ncbi.nlm.nih.gov/sites/entrez?Db=gene&Cmd=ShowDetailView&TermToSearch=835)*,* [*MORF4L1*](http://www.ncbi.nlm.nih.gov/sites/entrez?Db=gene&Cmd=ShowDetailView&TermToSearch=10933) | **Unique expression in hESCs** | [*RPS6KA5*](http://www.ncbi.nlm.nih.gov/sites/entrez?Db=gene&Cmd=ShowDetailView&TermToSearch=9252)*,* [*ARHGAP26*](http://www.ncbi.nlm.nih.gov/sites/entrez?Db=gene&Cmd=ShowDetailView&TermToSearch=23092)*,* [*BMPR2*](http://www.ncbi.nlm.nih.gov/sites/entrez?Db=gene&Cmd=ShowDetailView&TermToSearch=659)*,* [*MAP3K9*](http://www.ncbi.nlm.nih.gov/sites/entrez?Db=gene&Cmd=ShowDetailView&TermToSearch=4293)*,* [*RACGAP1*](http://www.ncbi.nlm.nih.gov/sites/entrez?Db=gene&Cmd=ShowDetailView&TermToSearch=29127)*,* [*MAP3K5*](http://www.ncbi.nlm.nih.gov/sites/entrez?Db=gene&Cmd=ShowDetailView&TermToSearch=4217)*,* [*RHOC*](http://www.ncbi.nlm.nih.gov/sites/entrez?Db=gene&Cmd=ShowDetailView&TermToSearch=389)*,* [*SMAD5*](http://www.ncbi.nlm.nih.gov/sites/entrez?Db=gene&Cmd=ShowDetailView&TermToSearch=4090)*, MAP3K12, MAP3K11* |
| hsa-miR-19a | 4.08 | 4.028E-100 | *RBBP8, PAK6, CCNT2, CDC2L5, CDC10, WEE1, CCND2, CCPG1* | *SHARP, SMARCA2, p300/CBP, SMARCD2, ARID1A* | *ANXA7* | *JAK1, OSM* | *LRP2, BMPR2, RHEBL1, ITPKB, RAB34, IGF2R, KRAS2, PLCB1, RAB18, INHBB* |
| hsa-miR-574-3p | 4.03 | 6.962E-136 | [*CNNM3*](http://www.ncbi.nlm.nih.gov/sites/entrez?Db=gene&Cmd=ShowDetailView&TermToSearch=26505) | [*METTL8*](http://www.ncbi.nlm.nih.gov/sites/entrez?Db=gene&Cmd=ShowDetailView&TermToSearch=79828)*,* [*ARID4B*](http://www.ncbi.nlm.nih.gov/sites/entrez?Db=gene&Cmd=ShowDetailView&TermToSearch=51742)*,*  [*NDST3*](http://www.ncbi.nlm.nih.gov/sites/entrez?Db=gene&Cmd=ShowDetailView&TermToSearch=9348)*,* [*METTL9*](http://www.ncbi.nlm.nih.gov/sites/entrez?Db=gene&Cmd=ShowDetailView&TermToSearch=51108) | *-* | *-* | [*SMAD3*](http://www.ncbi.nlm.nih.gov/sites/entrez?Db=gene&Cmd=ShowDetailView&TermToSearch=4088) |
| **hsa-miR-302c*** | 4.01 | 3.116E-13 | *-* | *CHD6* | *-* | **Unique expression in hESCs** | *LEF1* |
| hsa-miR-1308 | 3.98 | 5.455E-25 | *-* | *-* | *-* | [*GABRB3*](http://www.ncbi.nlm.nih.gov/sites/entrez?Db=gene&Cmd=ShowDetailView&TermToSearch=2562) | *-* |
| hsa-miR-1915 | 3.97 | 1.336E-116 | *-* | *-* | *-* | *-* | *-* |
| **hsa-miR-124** | 3.93 | 2.141E-07 | *CDK6, NEK4* | *EZH2, LMNA* | *-* | - | *-* |
| hsa-miR-183 | 3.87 | 9.323E-10 | *NEK9, CCND2* | *-* | *PDCD4* | - | *PLCB4, LRP6, MAP3K4, ARHGAP21, IRS1* |
| **hsa-miR-363** | 3.80 | 6.784E-127 | [*CDC27*](http://www.ncbi.nlm.nih.gov/sites/entrez?Db=gene&Cmd=ShowDetailView&TermToSearch=996)*,* [*CCNC*](http://www.ncbi.nlm.nih.gov/sites/entrez?Db=gene&Cmd=ShowDetailView&TermToSearch=892)*,* [*CCNJL*](http://www.ncbi.nlm.nih.gov/sites/entrez?Db=gene&Cmd=ShowDetailView&TermToSearch=79616)*,* [*CDKN1C*](http://www.ncbi.nlm.nih.gov/sites/entrez?Db=gene&Cmd=ShowDetailView&TermToSearch=1028)*,* [*WRNIP1*](http://www.ncbi.nlm.nih.gov/sites/entrez?Db=gene&Cmd=ShowDetailView&TermToSearch=56897) | *NIPBL,* [*ARID1B*](http://www.ncbi.nlm.nih.gov/sites/entrez?Db=gene&Cmd=ShowDetailView&TermToSearch=57492) | *-* | KLF4 | [*PIP5K3*](http://www.ncbi.nlm.nih.gov/sites/entrez?Db=gene&Cmd=ShowDetailView&TermToSearch=200576)*,* [*RGS17*](http://www.ncbi.nlm.nih.gov/sites/entrez?Db=gene&Cmd=ShowDetailView&TermToSearch=26575)*,* [*CAMK2A*](http://www.ncbi.nlm.nih.gov/sites/entrez?Db=gene&Cmd=ShowDetailView&TermToSearch=815)*,* [*PTGER4*](http://www.ncbi.nlm.nih.gov/sites/entrez?Db=gene&Cmd=ShowDetailView&TermToSearch=5734)*,* [*BMPR2*](http://www.ncbi.nlm.nih.gov/sites/entrez?Db=gene&Cmd=ShowDetailView&TermToSearch=659)*,* [*FZD10*](http://www.ncbi.nlm.nih.gov/sites/entrez?Db=gene&Cmd=ShowDetailView&TermToSearch=11211)*,* [*IRS2*](http://www.ncbi.nlm.nih.gov/sites/entrez?Db=gene&Cmd=ShowDetailView&TermToSearch=8660)*,* [*SMAD7*](http://www.ncbi.nlm.nih.gov/sites/entrez?Db=gene&Cmd=ShowDetailView&TermToSearch=4092)*,* [*NOTCH1*](http://www.ncbi.nlm.nih.gov/sites/entrez?Db=gene&Cmd=ShowDetailView&TermToSearch=4851) |
| hsa-miR-1260 | 3.76 | 1.026E-12 | *-* | [*MECP2*](http://www.ncbi.nlm.nih.gov/sites/entrez?Db=gene&Cmd=ShowDetailView&TermToSearch=4204)*,* [*JMJD3*](http://www.ncbi.nlm.nih.gov/sites/entrez?Db=gene&Cmd=ShowDetailView&TermToSearch=23135) | *-* | *-* | [*CSNK2B*](http://www.ncbi.nlm.nih.gov/sites/entrez?Db=gene&Cmd=ShowDetailView&TermToSearch=1460)*,* [*WNT10B*](http://www.ncbi.nlm.nih.gov/sites/entrez?Db=gene&Cmd=ShowDetailView&TermToSearch=7480)*,* [*RPS6KA3*](http://www.ncbi.nlm.nih.gov/sites/entrez?Db=gene&Cmd=ShowDetailView&TermToSearch=6197)*,* [*RACGAP1*](http://www.ncbi.nlm.nih.gov/sites/entrez?Db=gene&Cmd=ShowDetailView&TermToSearch=29127)*,* [*FZD4*](http://www.ncbi.nlm.nih.gov/sites/entrez?Db=gene&Cmd=ShowDetailView&TermToSearch=8322) |
| hsa-miR-301a | 3.74 | 6.179E-21 | [*p15RS*](http://www.ncbi.nlm.nih.gov/sites/entrez?Db=gene&Cmd=ShowDetailView&TermToSearch=55197) | [*MECP2*](http://www.ncbi.nlm.nih.gov/sites/entrez?Db=gene&Cmd=ShowDetailView&TermToSearch=4204)*,* [*SBF2*](http://www.ncbi.nlm.nih.gov/sites/entrez?Db=gene&Cmd=ShowDetailView&TermToSearch=81846) | *-* | [*ACVR1*](http://www.ncbi.nlm.nih.gov/sites/entrez?Db=gene&Cmd=ShowDetailView&TermToSearch=90)*,* [*TGFBR2*](http://www.ncbi.nlm.nih.gov/sites/entrez?Db=gene&Cmd=ShowDetailView&TermToSearch=7048) | [*RAP2C*](http://www.ncbi.nlm.nih.gov/sites/entrez?Db=gene&Cmd=ShowDetailView&TermToSearch=57826)*,* [*PIP5K3*](http://www.ncbi.nlm.nih.gov/sites/entrez?Db=gene&Cmd=ShowDetailView&TermToSearch=200576)*,* [*PIK3C2A*](http://www.ncbi.nlm.nih.gov/sites/entrez?Db=gene&Cmd=ShowDetailView&TermToSearch=5286)*, FRZB,* [*ARHGAP12*](http://www.ncbi.nlm.nih.gov/sites/entrez?Db=gene&Cmd=ShowDetailView&TermToSearch=94134)*, LRP6,* [*BMPR2*](http://www.ncbi.nlm.nih.gov/sites/entrez?Db=gene&Cmd=ShowDetailView&TermToSearch=659)*,* [*ARHGAP24*](http://www.ncbi.nlm.nih.gov/sites/entrez?Db=gene&Cmd=ShowDetailView&TermToSearch=83478)*, lRP8* |
| **hsa-miR-302b*** | 3.71 | 3.965E-10 | *-* | *MECP2, WT1, ARID4B* | *ING3, PCDC10* | **DICER1**  **Unique expression in hESCs** | *-* |
| hsa-miR-574-5p | 3.66 | 1.430E-98 | *-* | *-* | *-* | - | [*IHH*](http://www.ncbi.nlm.nih.gov/sites/entrez?Db=gene&Cmd=ShowDetailView&TermToSearch=3549)*, LIN7C* |
| hsa-miR-17 | 3.58 | 2.003E-10 | [*RBL2*](http://www.ncbi.nlm.nih.gov/sites/entrez?Db=gene&Cmd=ShowDetailView&TermToSearch=5934)*,* [*CDC40*](http://www.ncbi.nlm.nih.gov/sites/entrez?Db=gene&Cmd=ShowDetailView&TermToSearch=51362)*,* [*CCNG2*](http://www.ncbi.nlm.nih.gov/sites/entrez?Db=gene&Cmd=ShowDetailView&TermToSearch=901)*, p21* | [*ARID4B*](http://www.ncbi.nlm.nih.gov/sites/entrez?Db=gene&Cmd=ShowDetailView&TermToSearch=51742) | [*PDCD1LG2*](http://www.ncbi.nlm.nih.gov/sites/entrez?Db=gene&Cmd=ShowDetailView&TermToSearch=80380) | [*FRS2*](http://www.ncbi.nlm.nih.gov/sites/entrez?Db=gene&Cmd=ShowDetailView&TermToSearch=10818)*, JAK1, OSM* | [*GPR6*](http://www.ncbi.nlm.nih.gov/sites/entrez?Db=gene&Cmd=ShowDetailView&TermToSearch=2830)*,* [*MAP3K2*](http://www.ncbi.nlm.nih.gov/sites/entrez?Db=gene&Cmd=ShowDetailView&TermToSearch=10746)*,* [*ARHGAP26*](http://www.ncbi.nlm.nih.gov/sites/entrez?Db=gene&Cmd=ShowDetailView&TermToSearch=23092)*,* [*ARHGAP12*](http://www.ncbi.nlm.nih.gov/sites/entrez?Db=gene&Cmd=ShowDetailView&TermToSearch=94134)*,* [*RAB8B*](http://www.ncbi.nlm.nih.gov/sites/entrez?Db=gene&Cmd=ShowDetailView&TermToSearch=51762)*,* [*MAP3K9*](http://www.ncbi.nlm.nih.gov/sites/entrez?Db=gene&Cmd=ShowDetailView&TermToSearch=4293)*,* [*ARHGEF10*](http://www.ncbi.nlm.nih.gov/sites/entrez?Db=gene&Cmd=ShowDetailView&TermToSearch=9639)*,* [*RASL11B*](http://www.ncbi.nlm.nih.gov/sites/entrez?Db=gene&Cmd=ShowDetailView&TermToSearch=65997)*,* [*MAPK1*](http://www.ncbi.nlm.nih.gov/sites/entrez?Db=gene&Cmd=ShowDetailView&TermToSearch=5594)*,* [*RHOC*](http://www.ncbi.nlm.nih.gov/sites/entrez?Db=gene&Cmd=ShowDetailView&TermToSearch=389) |
| kshv-miR-K12-12 | 3.55 | 2.956E-08 | *RBL2* | *DNMT3A, DNMT3B* | *-* | - | *-* |
| hsv1-miR-H6 | 3.50 | 1.755E-44 | *-* | *-* | *-* | - | *-* |
| hsa-miR-1281 | 3.50 | 1.504E-54 | *-* | *HDAC4* | *-* | - | *-* |
| hsa-miR-1825 | 3.47 | 2.934E-74 | [*CCNK*](http://www.ncbi.nlm.nih.gov/sites/entrez?Db=gene&Cmd=ShowDetailView&TermToSearch=8812)*,* [*CDCA7L*](http://www.ncbi.nlm.nih.gov/sites/entrez?Db=gene&Cmd=ShowDetailView&TermToSearch=55536) | [*SMARCAD1*](http://www.ncbi.nlm.nih.gov/sites/entrez?Db=gene&Cmd=ShowDetailView&TermToSearch=56916)*,* [*SMARCA4*](http://www.ncbi.nlm.nih.gov/sites/entrez?Db=gene&Cmd=ShowDetailView&TermToSearch=6597) | *-* | [*ACVR2A*](http://www.ncbi.nlm.nih.gov/sites/entrez?Db=gene&Cmd=ShowDetailView&TermToSearch=92)*,* [*ACVR1B*](http://www.ncbi.nlm.nih.gov/sites/entrez?Db=gene&Cmd=ShowDetailView&TermToSearch=91) | [*IKBKB*](http://www.ncbi.nlm.nih.gov/sites/entrez?Db=gene&Cmd=ShowDetailView&TermToSearch=3551)*,* [*FZD4*](http://www.ncbi.nlm.nih.gov/sites/entrez?Db=gene&Cmd=ShowDetailView&TermToSearch=8322)*,FZD6,* [*MAP3K11*](http://www.ncbi.nlm.nih.gov/sites/entrez?Db=gene&Cmd=ShowDetailView&TermToSearch=4296)*,* [*ARHGEF12*](http://www.ncbi.nlm.nih.gov/sites/entrez?Db=gene&Cmd=ShowDetailView&TermToSearch=23365)*,* [*ARHGAP19*](http://www.ncbi.nlm.nih.gov/sites/entrez?Db=gene&Cmd=ShowDetailView&TermToSearch=84986) |
| hsa-miR-92a | 3.46 | 8.516E-29 | *RAD21, CDC27, p57, p35* | *NIPBL, ARID1B* | *-* | [*S1PR1*](http://www.ncbi.nlm.nih.gov/sites/entrez?Db=gene&Cmd=ShowDetailView&TermToSearch=1901) | *MAP2K4, PIK3R3, RGS17, CAMK2A, RGS3, BMPR2, SMAD7, NOTCH1* |
| hsa-miR-663 | 3.43 | 1.121E-35 | *-* | *-* | *-* | *-* | [*JUNB*](http://www.ncbi.nlm.nih.gov/sites/entrez?Db=gene&Cmd=ShowDetailView&TermToSearch=3726)*,* [*MAPK8IP3*](http://www.ncbi.nlm.nih.gov/sites/entrez?Db=gene&Cmd=ShowDetailView&TermToSearch=23162)*,* [*TGFB1*](http://www.ncbi.nlm.nih.gov/sites/entrez?Db=gene&Cmd=ShowDetailView&TermToSearch=7040) |
| hsa-miR-130b | 3.35 | 1.206E-65 | *CDK11, CDC14A, TP53INP1, SKP1A, PAK6* | *MECP2* | *-* | *ACVR1, LIN28* | *RPS6KA5, SMAD5, WNT1, WNT2B, MAP3K9, RAB5A, MAP3K14, WNT10B, NOGGIN,* |
| hsa-miR-1274a | 3.33 | 3.299E-28 | [*PAK6*](http://www.ncbi.nlm.nih.gov/sites/entrez?Db=gene&Cmd=ShowDetailView&TermToSearch=56924)*,* [*CCNJ*](http://www.ncbi.nlm.nih.gov/sites/entrez?Db=gene&Cmd=ShowDetailView&TermToSearch=54619) | [*JHDM1D*](http://www.ncbi.nlm.nih.gov/sites/entrez?Db=gene&Cmd=ShowDetailView&TermToSearch=80853)*,* [*ARID3A*](http://www.ncbi.nlm.nih.gov/sites/entrez?Db=gene&Cmd=ShowDetailView&TermToSearch=1820) | [*PDCD4*](http://www.ncbi.nlm.nih.gov/sites/entrez?Db=gene&Cmd=ShowDetailView&TermToSearch=27250) | *-* | [*BMPR1B*](http://www.ncbi.nlm.nih.gov/sites/entrez?Db=gene&Cmd=ShowDetailView&TermToSearch=658)*,* [*RAB14*](http://www.ncbi.nlm.nih.gov/sites/entrez?Db=gene&Cmd=ShowDetailView&TermToSearch=51552)*, FZD4* |
| hsa-miR-1207-5p | 3.30 | 4.093E-125 | [*CWF19L1*](http://www.ncbi.nlm.nih.gov/sites/entrez?Db=gene&Cmd=ShowDetailView&TermToSearch=55280) | [*SMARCD1*](http://www.ncbi.nlm.nih.gov/sites/entrez?Db=gene&Cmd=ShowDetailView&TermToSearch=6602)*,* [*METTL7B*](http://www.ncbi.nlm.nih.gov/sites/entrez?Db=gene&Cmd=ShowDetailView&TermToSearch=196410)*,* [*SET*](http://www.ncbi.nlm.nih.gov/sites/entrez?Db=gene&Cmd=ShowDetailView&TermToSearch=6418)*,* [*MYST2*](http://www.ncbi.nlm.nih.gov/sites/entrez?Db=gene&Cmd=ShowDetailView&TermToSearch=11143)*,* [*MBD6*](http://www.ncbi.nlm.nih.gov/sites/entrez?Db=gene&Cmd=ShowDetailView&TermToSearch=114785)*,* [*MECP2*](http://www.ncbi.nlm.nih.gov/sites/entrez?Db=gene&Cmd=ShowDetailView&TermToSearch=4204) | *-* | [*GABRB3*](http://www.ncbi.nlm.nih.gov/sites/entrez?Db=gene&Cmd=ShowDetailView&TermToSearch=2556)*,* [*IGF1*](http://www.ncbi.nlm.nih.gov/sites/entrez?Db=gene&Cmd=ShowDetailView&TermToSearch=3479)*,* | [*MAP3K14*](http://www.ncbi.nlm.nih.gov/sites/entrez?Db=gene&Cmd=ShowDetailView&TermToSearch=9020)*,* [*IGF1*](http://www.ncbi.nlm.nih.gov/sites/entrez?Db=gene&Cmd=ShowDetailView&TermToSearch=3479)*,* [*FGFR1*](http://www.ncbi.nlm.nih.gov/sites/entrez?Db=gene&Cmd=ShowDetailView&TermToSearch=2260) |
| hsa-miR-494 | 3.29 | 5.030E-40 | [*CCNT2*](http://www.ncbi.nlm.nih.gov/sites/entrez?Db=gene&Cmd=ShowDetailView&TermToSearch=905)*,* [*P15RS*](http://www.ncbi.nlm.nih.gov/sites/entrez?Db=gene&Cmd=ShowDetailView&TermToSearch=55197)*,* [*CNNM3*](http://www.ncbi.nlm.nih.gov/sites/entrez?Db=gene&Cmd=ShowDetailView&TermToSearch=26505)*, CCND2* | [*SAP18*](http://www.ncbi.nlm.nih.gov/sites/entrez?Db=gene&Cmd=ShowDetailView&TermToSearch=10284)*, MBD5,* [*SMARCE1*](http://www.ncbi.nlm.nih.gov/sites/entrez?Db=gene&Cmd=ShowDetailView&TermToSearch=6605)*,* [*SETD7*](http://www.ncbi.nlm.nih.gov/sites/entrez?Db=gene&Cmd=ShowDetailView&TermToSearch=80854)*,* [*JARID1C*](http://www.ncbi.nlm.nih.gov/sites/entrez?Db=gene&Cmd=ShowDetailView&TermToSearch=8242) | [*MORF4L2*](http://www.ncbi.nlm.nih.gov/sites/entrez?Db=gene&Cmd=ShowDetailView&TermToSearch=9643)*,* [*SIRT1*](http://www.ncbi.nlm.nih.gov/sites/entrez?Db=gene&Cmd=ShowDetailView&TermToSearch=23411) | *SOCS6,* [*ACVR1C*](http://www.ncbi.nlm.nih.gov/sites/entrez?Db=gene&Cmd=ShowDetailView&TermToSearch=130399)*,* [*FGFR2*](http://www.ncbi.nlm.nih.gov/sites/entrez?Db=gene&Cmd=ShowDetailView&TermToSearch=2263)*, LIF* | [*BMPR2*](http://www.ncbi.nlm.nih.gov/sites/entrez?Db=gene&Cmd=ShowDetailView&TermToSearch=659)*,* [*ARHGAP5*](http://www.ncbi.nlm.nih.gov/sites/entrez?Db=gene&Cmd=ShowDetailView&TermToSearch=394)*,* [*CSNK1G1*](http://www.ncbi.nlm.nih.gov/sites/entrez?Db=gene&Cmd=ShowDetailView&TermToSearch=53944)*,* [*PTEN*](http://www.ncbi.nlm.nih.gov/sites/entrez?Db=gene&Cmd=ShowDetailView&TermToSearch=5728)*,* [*ROCK1*](http://www.ncbi.nlm.nih.gov/sites/entrez?Db=gene&Cmd=ShowDetailView&TermToSearch=6093)*,* [*RAB40B*](http://www.ncbi.nlm.nih.gov/sites/entrez?Db=gene&Cmd=ShowDetailView&TermToSearch=10966)*,* [*HHIP*](http://www.ncbi.nlm.nih.gov/sites/entrez?Db=gene&Cmd=ShowDetailView&TermToSearch=64399)*,* [*ARHGAP26*](http://www.ncbi.nlm.nih.gov/sites/entrez?Db=gene&Cmd=ShowDetailView&TermToSearch=23092)*,* [*PIK3R3*](http://www.ncbi.nlm.nih.gov/sites/entrez?Db=gene&Cmd=ShowDetailView&TermToSearch=8503)*, GREM2* |
| hsa-miR-1181 | 3.18 | 4.488E-30 | - | - | - | - | - |
| ebv-miR-BART13 | 3.02 | 5.546E-11 | *-* | *-* | *-* | - | *-* |

**Supplemental Table 1.** Summary of miRNAs that are significantly up-regulated in iPSCs compared to DFs. For miRNAs target prediction and annotation to specific pathways, our own curation analysis and a combination of distinct software tools was used: TargetScan (http://www.targetscan.org/), miRanda (http://www.microrna.org/), Pictar (http://www.pictar.org/), Pubmed (http://www.ncbi.nlm.nih.gov/pubmed/). The miRNA that are highlighted in blue have been shown to contain conserved binding sites for *OCT4, SOX2, NANOG* and *TCF3*, whilst the miRNAs highlighted in purple have been shown to contain conserved binding sire for *OCT4, SOX2, NANOG, TCF3* and *PcG* complex. Analysis of miRNA seed sequences shows five clusters that share very similar seed sequences and may indicate common transcriptional targets (**cluster 1**: hsa-miR-302a, hsa-miR-302c, hsa-miR-302a*, hsa-miR-302d, hsa-miR-302b, hsa-miR-302c*, hsa-miR-20b, hsa-miR-17; **cluster 2**: hsa-miR-367, hsa-miR-363, hsa-miR-92a; **cluster 3**: hsa-miR-301a and hsa-miR-130b; **cluster 4**: hsa-miR-574-3p, hsa-miR-574-5p; **cluster 5**: hsa-miR-18b, hsa-miR-18a).
