# Supplementary material for: Brief Report: Inhibition of miR‐145 Enhances Reprogramming of Human Dermal Fibroblasts to Induced Pluripotent Stem Cells
Source: Stem Cells. 2015 Oct 9;34(1):246–51. doi: 10.1002/stem.2220 (PMC4982107; doi:10.1002/stem.2220)
Supplement: Supplementary file 4 — Supporting Information [file STEM-34-246-s004.docx]

**Supplemental Table 2**

| **miRNA species** | **Fold change [log] upregulation in human dermal fibroblasts versus iPSCs** | **Adjusted p-value** | **Predicted target gene involved in cell cycle regulation and DNA repair** | **Predicted target gene involved in chromatin modelling and transcription** | **Predicted target gene involved in cell proliferation or apoptosis** | **Predicted target gene involved in maintenance of pluripotency** | **Predicted target gene involved in key signalling pathways** |
| --- | --- | --- | --- | --- | --- | --- | --- |
| hsa-miR-451 | 11.58 | 1.455E-175 | *p19* | *-* | *-* | *-* | *-* |
| hsa-miR-516b | 7.38 | 1.048E-115 | *CCNG1, CDK6* | *TGIF2* | *-* | *ACVR2B* | *MAPK4* |
| hsa-miR-517b | 6.52 | 1.148E-43 | *-* | *-* | *-* | *-* | *WNT4* |
| hsa-miR-517a | 6.37 | 5.356E-25 | *-* | *-* | *-* | *-* | *WNT4* |
| hsa-miR-522 | 6.31 | 4.116E-98 | *FANCD2* | *-* | *-* | *-* | *RSU1* |
| hsa-miR-518e | 6.20 | 1.643E-85 | *CCNL2* | *-* | *-* | *-* | *-* |
| hsa-miR-144 | 6.14 | 8.293E-82 | *CCNT2* | *ARID1A, SMARCA1, TGIF* | *-* | *-* | *MAP3K4* |
| hsa-miR-376c | 6.08 | 1.713E-112 | *-* | *ARID4A, JMJD1C* | *BMI1* | *-* | *-* |
| hsa-miR-516a-5p | 6.06 | 2.454E-72 | *-* | *-* | *-* | *-* | *-* |
| hsa-miR-526b | 5.90 | 4.482E-53 | *CDK6*  *PAK1* | *-* | *-* | *TGFβR1* | *MAP3K7, TGFβR1* |
| hsa-miR-519d | 5.82 | 1.350E-59 | *p130, CDC40, CCNG2, CDC23, p21* | [*ARID4B*](http://www.ncbi.nlm.nih.gov/sites/entrez?Db=gene&Cmd=ShowDetailView&TermToSearch=51742)*, ARID4A* | [*PDCD1LG2*](http://www.ncbi.nlm.nih.gov/sites/entrez?Db=gene&Cmd=ShowDetailView&TermToSearch=80380)*, AEN* | [*STAT3*](http://www.ncbi.nlm.nih.gov/sites/entrez?Db=gene&Cmd=ShowDetailView&TermToSearch=6774) | [*RAB22A*](http://www.ncbi.nlm.nih.gov/sites/entrez?Db=gene&Cmd=ShowDetailView&TermToSearch=57403)*,* [*ARHGAP12*](http://www.ncbi.nlm.nih.gov/sites/entrez?Db=gene&Cmd=ShowDetailView&TermToSearch=94134)*,* [*BMPR2*](http://www.ncbi.nlm.nih.gov/sites/entrez?Db=gene&Cmd=ShowDetailView&TermToSearch=659)*,* [*RASSF2*](http://www.ncbi.nlm.nih.gov/sites/entrez?Db=gene&Cmd=ShowDetailView&TermToSearch=9770)*,* [*MAP3K9*](http://www.ncbi.nlm.nih.gov/sites/entrez?Db=gene&Cmd=ShowDetailView&TermToSearch=4293)*, MAPK1* |
| hsa-miR-521 | 5.76 | 1.822E-68 | *-* | *-* | *-* | *-* | *-* |
| hsa-miR-1323 | 5.46 | 2.431E-32 | *SMC6,* [*CDK5R1*](http://www.ncbi.nlm.nih.gov/sites/entrez?Db=gene&Cmd=ShowDetailView&TermToSearch=8851)*,* [*PAK7*](http://www.ncbi.nlm.nih.gov/sites/entrez?Db=gene&Cmd=ShowDetailView&TermToSearch=57144)*,* [*CNNM4*](http://www.ncbi.nlm.nih.gov/sites/entrez?Db=gene&Cmd=ShowDetailView&TermToSearch=26504) | [*JMJD2C*](http://www.ncbi.nlm.nih.gov/sites/entrez?Db=gene&Cmd=ShowDetailView&TermToSearch=23081)*,* [*JHDM1D*](http://www.ncbi.nlm.nih.gov/sites/entrez?Db=gene&Cmd=ShowDetailView&TermToSearch=80853)*,* [*MECP2*](http://www.ncbi.nlm.nih.gov/sites/entrez?Db=gene&Cmd=ShowDetailView&TermToSearch=4204) | [*PDCD7*](http://www.ncbi.nlm.nih.gov/sites/entrez?Db=gene&Cmd=ShowDetailView&TermToSearch=10081) | *-* | [*PRKAA2*](http://www.ncbi.nlm.nih.gov/sites/entrez?Db=gene&Cmd=ShowDetailView&TermToSearch=5563)*,* [*JAG1*](http://www.ncbi.nlm.nih.gov/sites/entrez?Db=gene&Cmd=ShowDetailView&TermToSearch=182)*, FGF5,* [*PRKACA*](http://www.ncbi.nlm.nih.gov/sites/entrez?Db=gene&Cmd=ShowDetailView&TermToSearch=5566)*,* [*PIK3R1*](http://www.ncbi.nlm.nih.gov/sites/entrez?Db=gene&Cmd=ShowDetailView&TermToSearch=5295)*,* [*MAPK1*](http://www.ncbi.nlm.nih.gov/sites/entrez?Db=gene&Cmd=ShowDetailView&TermToSearch=5594) |
| hsa-miR-517c | 5.28 | 6.722E-30 | *-* | *-* | *-* | *-* | *WNT4* |
| hsa-miR-376a | 5.13 | 6.406E-48 | *CCNJ, p35* | *HDAC9* | *-* | *FGFR1, TGFBR1* | *BMPR2, SOCS4* |
| hsa-miR-515-5p | 5.11 | 1.747E-41 | [*TP53INP2*](http://www.ncbi.nlm.nih.gov/sites/entrez?Db=gene&Cmd=ShowDetailView&TermToSearch=58476) | [*JHDM1D*](http://www.ncbi.nlm.nih.gov/sites/entrez?Db=gene&Cmd=ShowDetailView&TermToSearch=80853)*,* [*ARID3B*](http://www.ncbi.nlm.nih.gov/sites/entrez?Db=gene&Cmd=ShowDetailView&TermToSearch=10620) | [*API5*](http://www.ncbi.nlm.nih.gov/sites/entrez?Db=gene&Cmd=ShowDetailView&TermToSearch=8539) | *FGFR2,* [*ACVR2B*](http://www.ncbi.nlm.nih.gov/sites/entrez?Db=gene&Cmd=ShowDetailView&TermToSearch=93) | *NOTCH2, FGF12,* [*BMP8B*](http://www.ncbi.nlm.nih.gov/sites/entrez?Db=gene&Cmd=ShowDetailView&TermToSearch=656)*,* [*PIK3CG*](http://www.ncbi.nlm.nih.gov/sites/entrez?Db=gene&Cmd=ShowDetailView&TermToSearch=5294)*,* [*MAP4K4*](http://www.ncbi.nlm.nih.gov/sites/entrez?Db=gene&Cmd=ShowDetailView&TermToSearch=9448)*,* [*IKBKB*](http://www.ncbi.nlm.nih.gov/sites/entrez?Db=gene&Cmd=ShowDetailView&TermToSearch=3551) |
| hsa-miR-518c* | 4.97 | 9.548E-37 | *-* | *-* | *-* | *-* | *-* |
| hsa-miR-223 | 4.93 | 1.199E-66 | *CCNT2,RASA1* | *MYST3* | *-* | *ACVR2, FGFR2* | *RPS6KB1, MAPK10, MAP3K2, RHOB, RRAS2, RGS1* |
| hsa-miR-377 | 4.90 | 1.717E-62 | *RASA1, MDM2, p27* | *ARID1A* | *XIAP, p63* | *-* | *KRAS2, JAK2* |
| hsa-miR-518e* | 4.81 | 4.366E-90 | *-* | *-* | *-* | *-* | *-* |
| hsa-miR-142-3p | 4.81 | 3.532E-59 | *-* | *-* | *MORF4L1* | *IL6* | *-* |
| hsa-miR-525-5p | 4.81 | 1.417E-39 | [*CNNM1*](http://www.ncbi.nlm.nih.gov/sites/entrez?Db=gene&Cmd=ShowDetailView&TermToSearch=26507)*,* [*CNNM3*](http://www.ncbi.nlm.nih.gov/sites/entrez?Db=gene&Cmd=ShowDetailView&TermToSearch=26505) | [*SMARCA1*](http://www.ncbi.nlm.nih.gov/sites/entrez?Db=gene&Cmd=ShowDetailView&TermToSearch=6594)*,* [*HDAC7*](http://www.ncbi.nlm.nih.gov/sites/entrez?Db=gene&Cmd=ShowDetailView&TermToSearch=51564)*,* [*METTL10*](http://www.ncbi.nlm.nih.gov/sites/entrez?Db=gene&Cmd=ShowDetailView&TermToSearch=399818) | [*NAIP*](http://www.ncbi.nlm.nih.gov/sites/entrez?Db=gene&Cmd=ShowDetailView&TermToSearch=4671) | *-* | [*RGS4*](http://www.ncbi.nlm.nih.gov/sites/entrez?Db=gene&Cmd=ShowDetailView&TermToSearch=5999)*,* [*PIK3R1*](http://www.ncbi.nlm.nih.gov/sites/entrez?Db=gene&Cmd=ShowDetailView&TermToSearch=5295)*, RHGEF3,* [*RGS6*](http://www.ncbi.nlm.nih.gov/sites/entrez?Db=gene&Cmd=ShowDetailView&TermToSearch=9628)*,* [*CAMK2A*](http://www.ncbi.nlm.nih.gov/sites/entrez?Db=gene&Cmd=ShowDetailView&TermToSearch=815) |
| hsa-miR-518a-3p | 4.71 | 1.110E-43 | *CCNL2* | *-* | *-* | *-* | *RPS6KA3* |
| hsa-miR-520h | 4.70 | 1.847E-49 | *p21, CCND2,* [*TP53INP1*](http://www.ncbi.nlm.nih.gov/sites/entrez?Db=gene&Cmd=ShowDetailView&TermToSearch=94241) | *HDAC4* | *-* | *LIN28B* | [*RND3*](http://www.ncbi.nlm.nih.gov/sites/entrez?Db=gene&Cmd=ShowDetailView&TermToSearch=390)*, BMP6, MAP3K9, JAK1, SMAD6, MAP3K8, SOCS6* |
| hsa-miR-518b | 4.67 | 5.166E-28 | *CCNL2* | *-* | *EGR1* | *-* | *-* |
| hsa-miR-495 | 4.54 | 7.660E-64 | *CDK6, p15, CDC14A, E2F2* | [*JMJD2A*](http://www.ncbi.nlm.nih.gov/sites/entrez?Db=gene&Cmd=ShowDetailView&TermToSearch=9682) | *GAS1, XIAP* | *DICER1, ACVR2B* | *MAPK10,* [*MAP3K7IP2*](http://www.ncbi.nlm.nih.gov/sites/entrez?Db=gene&Cmd=ShowDetailView&TermToSearch=23118)*, TCF4, IGF1* |
| hsa-miR-520a-5p | 4.51 | 1.455E-29 | [*SMC1A*](http://www.ncbi.nlm.nih.gov/sites/entrez?Db=gene&Cmd=ShowDetailView&TermToSearch=8243)*,* [*CNNM1*](http://www.ncbi.nlm.nih.gov/sites/entrez?Db=gene&Cmd=ShowDetailView&TermToSearch=26507)*,* [*CNNM3*](http://www.ncbi.nlm.nih.gov/sites/entrez?Db=gene&Cmd=ShowDetailView&TermToSearch=26505) | [*SMARCA1*](http://www.ncbi.nlm.nih.gov/sites/entrez?Db=gene&Cmd=ShowDetailView&TermToSearch=6594)*,* [*METTL10*](http://www.ncbi.nlm.nih.gov/sites/entrez?Db=gene&Cmd=ShowDetailView&TermToSearch=399818)*,* [*HDAC7*](http://www.ncbi.nlm.nih.gov/sites/entrez?Db=gene&Cmd=ShowDetailView&TermToSearch=51564) | *-* | [*IGF1R*](http://www.ncbi.nlm.nih.gov/sites/entrez?Db=gene&Cmd=ShowDetailView&TermToSearch=3480)*,* [*ACVR2B*](http://www.ncbi.nlm.nih.gov/sites/entrez?Db=gene&Cmd=ShowDetailView&TermToSearch=93) | [*RGS4*](http://www.ncbi.nlm.nih.gov/sites/entrez?Db=gene&Cmd=ShowDetailView&TermToSearch=5999)*,* [*PIK3R1*](http://www.ncbi.nlm.nih.gov/sites/entrez?Db=gene&Cmd=ShowDetailView&TermToSearch=5295)*, WNT4,* [*FZD9*](http://www.ncbi.nlm.nih.gov/sites/entrez?Db=gene&Cmd=ShowDetailView&TermToSearch=8326)*,* [*CALM2*](http://www.ncbi.nlm.nih.gov/sites/entrez?Db=gene&Cmd=ShowDetailView&TermToSearch=805)*, WNT3,* [*RASL10B*](http://www.ncbi.nlm.nih.gov/sites/entrez?Db=gene&Cmd=ShowDetailView&TermToSearch=91608) |
| hsa-miR-515-3p | 4.19 | 1.857E-47 | [*PDRG1*](http://www.ncbi.nlm.nih.gov/sites/entrez?Db=gene&Cmd=ShowDetailView&TermToSearch=81572)*,* [*SMC1A*](http://www.ncbi.nlm.nih.gov/sites/entrez?Db=gene&Cmd=ShowDetailView&TermToSearch=8243) | [*SMARCAD1*](http://www.ncbi.nlm.nih.gov/sites/entrez?Db=gene&Cmd=ShowDetailView&TermToSearch=56916)*,* [*MBD6*](http://www.ncbi.nlm.nih.gov/sites/entrez?Db=gene&Cmd=ShowDetailView&TermToSearch=114785) | [*DICER1*](http://www.ncbi.nlm.nih.gov/sites/entrez?Db=gene&Cmd=ShowDetailView&TermToSearch=23405) | *-* | [*RPS6KA3*](http://www.ncbi.nlm.nih.gov/sites/entrez?Db=gene&Cmd=ShowDetailView&TermToSearch=6197)*,* [*RABGAP1*](http://www.ncbi.nlm.nih.gov/sites/entrez?Db=gene&Cmd=ShowDetailView&TermToSearch=23637) *,*  *FGFR1* |
| hsa-miR-410 | 4.04 | 8.989E-74 | *CCNA2,ATRX* | *ARID2* | - | **Upregulated during ESC differentiation** | - |
| hsa-miR-518c | 4.03 | 5,605E-61 |  |  |  |  |  |
| hsa-miR-519b-3p | 3.80 | 2.663E-35 | [*CCNJ*](http://www.ncbi.nlm.nih.gov/sites/entrez?Db=gene&Cmd=ShowDetailView&TermToSearch=54619)*,* [*CDK2*](http://www.ncbi.nlm.nih.gov/sites/entrez?Db=gene&Cmd=ShowDetailView&TermToSearch=1017)*,* [*CAPRIN1*](http://www.ncbi.nlm.nih.gov/sites/entrez?Db=gene&Cmd=ShowDetailView&TermToSearch=4076)*,*  [*FANCM*](http://www.ncbi.nlm.nih.gov/sites/entrez?Db=gene&Cmd=ShowDetailView&TermToSearch=57697) | [*ARID4A*](http://www.ncbi.nlm.nih.gov/sites/entrez?Db=gene&Cmd=ShowDetailView&TermToSearch=5926)*,* [*SUV39H1*](http://www.ncbi.nlm.nih.gov/sites/entrez?Db=gene&Cmd=ShowDetailView&TermToSearch=6839) | [*ING1*](http://www.ncbi.nlm.nih.gov/sites/entrez?Db=gene&Cmd=ShowDetailView&TermToSearch=3621) | *-* | [*ARHGAP12*](http://www.ncbi.nlm.nih.gov/sites/entrez?Db=gene&Cmd=ShowDetailView&TermToSearch=94134)*,* [*JAKMIP1*](http://www.ncbi.nlm.nih.gov/sites/entrez?Db=gene&Cmd=ShowDetailView&TermToSearch=152789)*,* [*RAB8A*](http://www.ncbi.nlm.nih.gov/sites/entrez?Db=gene&Cmd=ShowDetailView&TermToSearch=4218)*,* [*JAK1*](http://www.ncbi.nlm.nih.gov/sites/entrez?Db=gene&Cmd=ShowDetailView&TermToSearch=3716)*,* [*ARHGAP24*](http://www.ncbi.nlm.nih.gov/sites/entrez?Db=gene&Cmd=ShowDetailView&TermToSearch=83478)*,* [*BMPR2*](http://www.ncbi.nlm.nih.gov/sites/entrez?Db=gene&Cmd=ShowDetailView&TermToSearch=659)*,* [*ITPR1*](http://www.ncbi.nlm.nih.gov/sites/entrez?Db=gene&Cmd=ShowDetailView&TermToSearch=3708)*,* [*ARHGAP29*](http://www.ncbi.nlm.nih.gov/sites/entrez?Db=gene&Cmd=ShowDetailView&TermToSearch=9411)*,* [*SNIP1*](http://www.ncbi.nlm.nih.gov/sites/entrez?Db=gene&Cmd=ShowDetailView&TermToSearch=79753)*,* [*PRKAA1*](http://www.ncbi.nlm.nih.gov/sites/entrez?Db=gene&Cmd=ShowDetailView&TermToSearch=5562)*,* [*GPR158*](http://www.ncbi.nlm.nih.gov/sites/entrez?Db=gene&Cmd=ShowDetailView&TermToSearch=57512)*,* [*RPS6KA5*](http://www.ncbi.nlm.nih.gov/sites/entrez?Db=gene&Cmd=ShowDetailView&TermToSearch=9252)*, SMAD7,* [*RPS6KA3*](http://www.ncbi.nlm.nih.gov/sites/entrez?Db=gene&Cmd=ShowDetailView&TermToSearch=6197)*,* [*ARHGAP1*](http://www.ncbi.nlm.nih.gov/sites/entrez?Db=gene&Cmd=ShowDetailView&TermToSearch=392)*,* [*MAP3K2*](http://www.ncbi.nlm.nih.gov/sites/entrez?Db=gene&Cmd=ShowDetailView&TermToSearch=10746)*,* [*TGFBR2*](http://www.ncbi.nlm.nih.gov/sites/entrez?Db=gene&Cmd=ShowDetailView&TermToSearch=7048)*,* [*ACVR1*](http://www.ncbi.nlm.nih.gov/sites/entrez?Db=gene&Cmd=ShowDetailView&TermToSearch=90) |
| hsa-miR-424 | 3.79 | 3.925E-44 | *CCNE1, WEE1,* [*CDCA4*](http://www.ncbi.nlm.nih.gov/sites/entrez?Db=gene&Cmd=ShowDetailView&TermToSearch=55038)*,* [*CDC27*](http://www.ncbi.nlm.nih.gov/sites/entrez?Db=gene&Cmd=ShowDetailView&TermToSearch=996)*,* [*CDC37L1*](http://www.ncbi.nlm.nih.gov/sites/entrez?Db=gene&Cmd=ShowDetailView&TermToSearch=55664)*,* [*CCND2*](http://www.ncbi.nlm.nih.gov/sites/entrez?Db=gene&Cmd=ShowDetailView&TermToSearch=894)*, p35,* [*CNNM2*](http://www.ncbi.nlm.nih.gov/sites/entrez?Db=gene&Cmd=ShowDetailView&TermToSearch=54805)*,* [*CAPRIN1*](http://www.ncbi.nlm.nih.gov/sites/entrez?Db=gene&Cmd=ShowDetailView&TermToSearch=4076)*,* [*CDC14A*](http://www.ncbi.nlm.nih.gov/sites/entrez?Db=gene&Cmd=ShowDetailView&TermToSearch=8556)*,* [*E2F3*](http://www.ncbi.nlm.nih.gov/sites/entrez?Db=gene&Cmd=ShowDetailView&TermToSearch=1871)*,* [*CCND1*](http://www.ncbi.nlm.nih.gov/sites/entrez?Db=gene&Cmd=ShowDetailView&TermToSearch=595)*,* [*CCND3*](http://www.ncbi.nlm.nih.gov/sites/entrez?Db=gene&Cmd=ShowDetailView&TermToSearch=896)*,* [*G0S2*](http://www.ncbi.nlm.nih.gov/sites/entrez?Db=gene&Cmd=ShowDetailView&TermToSearch=50486)*,* [*CCNJL*](http://www.ncbi.nlm.nih.gov/sites/entrez?Db=gene&Cmd=ShowDetailView&TermToSearch=79616)*,* [*CHEK1*](http://www.ncbi.nlm.nih.gov/sites/entrez?Db=gene&Cmd=ShowDetailView&TermToSearch=1111) | [*SETD3*](http://www.ncbi.nlm.nih.gov/sites/entrez?Db=gene&Cmd=ShowDetailView&TermToSearch=84193)*,* [*JARID2*](http://www.ncbi.nlm.nih.gov/sites/entrez?Db=gene&Cmd=ShowDetailView&TermToSearch=3720) | [*GHR*](http://www.ncbi.nlm.nih.gov/sites/entrez?Db=gene&Cmd=ShowDetailView&TermToSearch=2690)*,* [*PDCD6IP*](http://www.ncbi.nlm.nih.gov/sites/entrez?Db=gene&Cmd=ShowDetailView&TermToSearch=10015) | [*ACVR2B*](http://www.ncbi.nlm.nih.gov/sites/entrez?Db=gene&Cmd=ShowDetailView&TermToSearch=93)*,* | [*AXIN2*](http://www.ncbi.nlm.nih.gov/sites/entrez?Db=gene&Cmd=ShowDetailView&TermToSearch=8313)*,* [*MKNK1*](http://www.ncbi.nlm.nih.gov/sites/entrez?Db=gene&Cmd=ShowDetailView&TermToSearch=8569)*,* [*SMAD7*](http://www.ncbi.nlm.nih.gov/sites/entrez?Db=gene&Cmd=ShowDetailView&TermToSearch=4092)*, SMAD5,* [*MAP2K1*](http://www.ncbi.nlm.nih.gov/sites/entrez?Db=gene&Cmd=ShowDetailView&TermToSearch=5604)*,* [*MAP3K7IP3*](http://www.ncbi.nlm.nih.gov/sites/entrez?Db=gene&Cmd=ShowDetailView&TermToSearch=257397)*,* [*RPS6KA3*](http://www.ncbi.nlm.nih.gov/sites/entrez?Db=gene&Cmd=ShowDetailView&TermToSearch=6197)*,* [*RAP2C*](http://www.ncbi.nlm.nih.gov/sites/entrez?Db=gene&Cmd=ShowDetailView&TermToSearch=57826)*, RAF1,* [*SMURF2*](http://www.ncbi.nlm.nih.gov/sites/entrez?Db=gene&Cmd=ShowDetailView&TermToSearch=64750)*,* [*FGFR1*](http://www.ncbi.nlm.nih.gov/sites/entrez?Db=gene&Cmd=ShowDetailView&TermToSearch=2260) |
| hsa-miR-520g | 3.74 | 4.807E-36 | [*CCND2*](http://www.ncbi.nlm.nih.gov/sites/entrez?Db=gene&Cmd=ShowDetailView&TermToSearch=894)*,* [*WEE1*](http://www.ncbi.nlm.nih.gov/sites/entrez?Db=gene&Cmd=ShowDetailView&TermToSearch=7465)*,* [*FANCA*](http://www.ncbi.nlm.nih.gov/sites/entrez?Db=gene&Cmd=ShowDetailView&TermToSearch=94241) | *-* | [*TP53INP1*](http://www.ncbi.nlm.nih.gov/sites/entrez?Db=gene&Cmd=ShowDetailView&TermToSearch=94241) | [*LIN28B*](http://www.ncbi.nlm.nih.gov/sites/entrez?Db=gene&Cmd=ShowDetailView&TermToSearch=389421) | [*RND3*](http://www.ncbi.nlm.nih.gov/sites/entrez?Db=gene&Cmd=ShowDetailView&TermToSearch=390)*,* [*BMP6*](http://www.ncbi.nlm.nih.gov/sites/entrez?Db=gene&Cmd=ShowDetailView&TermToSearch=654)*,* [*PLCB1*](http://www.ncbi.nlm.nih.gov/sites/entrez?Db=gene&Cmd=ShowDetailView&TermToSearch=23236)*,* [*MAP3K9*](http://www.ncbi.nlm.nih.gov/sites/entrez?Db=gene&Cmd=ShowDetailView&TermToSearch=4293)*,* [*PRKCB1*](http://www.ncbi.nlm.nih.gov/sites/entrez?Db=gene&Cmd=ShowDetailView&TermToSearch=5579)*,* [*RACGAP1*](http://www.ncbi.nlm.nih.gov/sites/entrez?Db=gene&Cmd=ShowDetailView&TermToSearch=29127)*,* [*RAB5B*](http://www.ncbi.nlm.nih.gov/sites/entrez?Db=gene&Cmd=ShowDetailView&TermToSearch=5869)*, SMAD7,* [*ITPKB*](http://www.ncbi.nlm.nih.gov/sites/entrez?Db=gene&Cmd=ShowDetailView&TermToSearch=3707)*,* [*NLK*](http://www.ncbi.nlm.nih.gov/sites/entrez?Db=gene&Cmd=ShowDetailView&TermToSearch=51701)*, SMAD6,* [*MAP3K8*](http://www.ncbi.nlm.nih.gov/sites/entrez?Db=gene&Cmd=ShowDetailView&TermToSearch=1326)*, TGFBR3* |
| hsa-miR-517* | 3.74 | 9.587E-53 | *-* | *-* | *-* | *-* | *-* |
| hsa-miR-136* | 3.73 | 3.303E-81 | *-* | *JMJD1A, DNMT2* | *-* | *-* | *MNIK, RGS4, SMAD6, MAP2K1IP1* |
| hsa-miR-486-5p | 3.63 | 3.783E-58 | *-* | *ARID4B, HAT1,* [*SMARCD2*](http://www.ncbi.nlm.nih.gov/sites/entrez?Db=gene&Cmd=ShowDetailView&TermToSearch=6603)*, ARID1A* | *-* | *GABRB3,*  *SMAD2* | [*PIK3R1*](http://www.ncbi.nlm.nih.gov/sites/entrez?Db=gene&Cmd=ShowDetailView&TermToSearch=5295)*, IGF1,* |
| hsa-miR-30b | 3.55 | 3.694E-06 | *CCNT2, CDC37L1, CCNK* | *SHARP* | *SOCS1* | *LIN28, SOCS1,*  *SOC3* | *DGKZ, RAB32, RHEBL1, RASD1, JAG2, SRGAP3, PLCG1* |
| hsa-miR-224 | 3.50 | 3.639E-42 | *WEE1, p21, CDC4, CHES1* | *JMJD1A* | *API5* | *OSM* | *SMAD4, SMAD5* |
| hsa-miR-136 | 3.50 | 1.114E-44 | *CCPG1* | *JARID2* | *APAF1, GDF6, XIAP* | **Unique expression in hESCs** | *MAPK4* |
| hsa-miR-520d-3p | 3.47 | 1.265E-26 | [*CDCA7*](http://www.ncbi.nlm.nih.gov/sites/entrez?Db=gene&Cmd=ShowDetailView&TermToSearch=83879)*,* [*CCNJ*](http://www.ncbi.nlm.nih.gov/sites/entrez?Db=gene&Cmd=ShowDetailView&TermToSearch=54619)*,* [*PAK7*](http://www.ncbi.nlm.nih.gov/sites/entrez?Db=gene&Cmd=ShowDetailView&TermToSearch=57144)*,* [*E2F5*](http://www.ncbi.nlm.nih.gov/sites/entrez?Db=gene&Cmd=ShowDetailView&TermToSearch=1875)*,* [*RBL1*](http://www.ncbi.nlm.nih.gov/sites/entrez?Db=gene&Cmd=ShowDetailView&TermToSearch=5933)*,* [*CDC40*](http://www.ncbi.nlm.nih.gov/sites/entrez?Db=gene&Cmd=ShowDetailView&TermToSearch=51362)*,* [*CDC23*](http://www.ncbi.nlm.nih.gov/sites/entrez?Db=gene&Cmd=ShowDetailView&TermToSearch=8697)*,* [*TP53INP1*](http://www.ncbi.nlm.nih.gov/sites/entrez?Db=gene&Cmd=ShowDetailView&TermToSearch=94241)*,* [*CCND2*](http://www.ncbi.nlm.nih.gov/sites/entrez?Db=gene&Cmd=ShowDetailView&TermToSearch=894)*,* [*PAK2*](http://www.ncbi.nlm.nih.gov/sites/entrez?Db=gene&Cmd=ShowDetailView&TermToSearch=5062)*,* [*E2F2*](http://www.ncbi.nlm.nih.gov/sites/entrez?Db=gene&Cmd=ShowDetailView&TermToSearch=1870)*,* [*CNNM3*](http://www.ncbi.nlm.nih.gov/sites/entrez?Db=gene&Cmd=ShowDetailView&TermToSearch=26505)*,* [*CCND1*](http://www.ncbi.nlm.nih.gov/sites/entrez?Db=gene&Cmd=ShowDetailView&TermToSearch=595)*,* [*WEE1*](http://www.ncbi.nlm.nih.gov/sites/entrez?Db=gene&Cmd=ShowDetailView&TermToSearch=7465)*,* [*LATS2*](http://www.ncbi.nlm.nih.gov/sites/entrez?Db=gene&Cmd=ShowDetailView&TermToSearch=26524)*,* | [*MBD2*](http://www.ncbi.nlm.nih.gov/sites/entrez?Db=gene&Cmd=ShowDetailView&TermToSearch=8932)*,* [*ARID4A*](http://www.ncbi.nlm.nih.gov/sites/entrez?Db=gene&Cmd=ShowDetailView&TermToSearch=5926)*,* [*ARID5B*](http://www.ncbi.nlm.nih.gov/sites/entrez?Db=gene&Cmd=ShowDetailView&TermToSearch=84159)*,* [*HDAC4*](http://www.ncbi.nlm.nih.gov/sites/entrez?Db=gene&Cmd=ShowDetailView&TermToSearch=9759) | *-* | *FGFR2,* [*SMAD2*](http://www.ncbi.nlm.nih.gov/sites/entrez?Db=gene&Cmd=ShowDetailView&TermToSearch=4087) | [*RAB22A*](http://www.ncbi.nlm.nih.gov/sites/entrez?Db=gene&Cmd=ShowDetailView&TermToSearch=57403)*,* [*ARHGEF10*](http://www.ncbi.nlm.nih.gov/sites/entrez?Db=gene&Cmd=ShowDetailView&TermToSearch=9639)*,* [*FZD6*](http://www.ncbi.nlm.nih.gov/sites/entrez?Db=gene&Cmd=ShowDetailView&TermToSearch=8323)*,* [*RPS6KA3*](http://www.ncbi.nlm.nih.gov/sites/entrez?Db=gene&Cmd=ShowDetailView&TermToSearch=6197)*,* [*RND3*](http://www.ncbi.nlm.nih.gov/sites/entrez?Db=gene&Cmd=ShowDetailView&TermToSearch=390)*,* [*BMPR2*](http://www.ncbi.nlm.nih.gov/sites/entrez?Db=gene&Cmd=ShowDetailView&TermToSearch=659)*,* [*PLCL1*](http://www.ncbi.nlm.nih.gov/sites/entrez?Db=gene&Cmd=ShowDetailView&TermToSearch=5334)*,* [*MAP3K2*](http://www.ncbi.nlm.nih.gov/sites/entrez?Db=gene&Cmd=ShowDetailView&TermToSearch=10746)*,* [*MAP3K14*](http://www.ncbi.nlm.nih.gov/sites/entrez?Db=gene&Cmd=ShowDetailView&TermToSearch=9020)*,* [*ARHGAP24*](http://www.ncbi.nlm.nih.gov/sites/entrez?Db=gene&Cmd=ShowDetailView&TermToSearch=83478)*,* [*FGF9*](http://www.ncbi.nlm.nih.gov/sites/entrez?Db=gene&Cmd=ShowDetailView&TermToSearch=2254)*,* [*TGFBR2*](http://www.ncbi.nlm.nih.gov/sites/entrez?Db=gene&Cmd=ShowDetailView&TermToSearch=7048)*,* [*LEFTY1*](http://www.ncbi.nlm.nih.gov/sites/entrez?Db=gene&Cmd=ShowDetailView&TermToSearch=10637)*,* [*LEFTY2*](http://www.ncbi.nlm.nih.gov/sites/entrez?Db=gene&Cmd=ShowDetailView&TermToSearch=7044) |
| hsa-miR-26b | 3.45 | 1.939E-36 | *CCNJ, TP53INP, CDK6, CCNE2, CCNE1, NEK6, CDK2AP1, TP53INP2, PAK2, CCND2,* [*CCNL2*](http://www.ncbi.nlm.nih.gov/sites/entrez?Db=gene&Cmd=ShowDetailView&TermToSearch=81669)*,* [*CDC6*](http://www.ncbi.nlm.nih.gov/sites/entrez?Db=gene&Cmd=ShowDetailView&TermToSearch=990)*,* [*RB1*](http://www.ncbi.nlm.nih.gov/sites/entrez?Db=gene&Cmd=ShowDetailView&TermToSearch=5925)*,* [*CDK8*](http://www.ncbi.nlm.nih.gov/sites/entrez?Db=gene&Cmd=ShowDetailView&TermToSearch=1024)*,* [*CCNJL*](http://www.ncbi.nlm.nih.gov/sites/entrez?Db=gene&Cmd=ShowDetailView&TermToSearch=79616)*,* [*CCNJ*](http://www.ncbi.nlm.nih.gov/sites/entrez?Db=gene&Cmd=ShowDetailView&TermToSearch=79616)*, ATM* | *SETD8, HDAC9, ARID3A, DNMT3B* | *PDCD10* | *-* | *ARHGAP26, NLK, GSK3B, SMAD1, MAP3K2, ARHGAP21, PTGS2, PTEN, CAMK2A, RGS4, PIK3R3, MRAS, G3BP2,* [*MAPK6*](http://www.ncbi.nlm.nih.gov/sites/entrez?Db=gene&Cmd=ShowDetailView&TermToSearch=5597)*,* [*JAG1*](http://www.ncbi.nlm.nih.gov/sites/entrez?Db=gene&Cmd=ShowDetailView&TermToSearch=182)*,* [*MAP3K2*](http://www.ncbi.nlm.nih.gov/sites/entrez?Db=gene&Cmd=ShowDetailView&TermToSearch=10746)*, ACVR1C, TIEG* |
| hsa-miR-542-3p | 3.43 | 3.343E-46 | [*CAPRIN1*](http://www.ncbi.nlm.nih.gov/sites/entrez?Db=gene&Cmd=ShowDetailView&TermToSearch=4076)*, CCNM4* | *-* | *-* | [*ACVR1C*](http://www.ncbi.nlm.nih.gov/sites/entrez?Db=gene&Cmd=ShowDetailView&TermToSearch=130399) | [*PTEN*](http://www.ncbi.nlm.nih.gov/sites/entrez?Db=gene&Cmd=ShowDetailView&TermToSearch=5728)*,* [*RPS6KA4*](http://www.ncbi.nlm.nih.gov/sites/entrez?Db=gene&Cmd=ShowDetailView&TermToSearch=8986)*, BMP7,* [*MAP3K11*](http://www.ncbi.nlm.nih.gov/sites/entrez?Db=gene&Cmd=ShowDetailView&TermToSearch=4296) |
| hsa-miR-518f | 3.43 | 2.924E-35 | *CCNL2* | *-* | *EGR1* | *-* | *-* |
| hsa-miR-519c-3p | 3.36 | 2.602E-27 | [*CCNJ*](http://www.ncbi.nlm.nih.gov/sites/entrez?Db=gene&Cmd=ShowDetailView&TermToSearch=54619)*,* [*CDK2*](http://www.ncbi.nlm.nih.gov/sites/entrez?Db=gene&Cmd=ShowDetailView&TermToSearch=1017)*,* [*FANCM*](http://www.ncbi.nlm.nih.gov/sites/entrez?Db=gene&Cmd=ShowDetailView&TermToSearch=57697)*,* [*CAPRIN1*](http://www.ncbi.nlm.nih.gov/sites/entrez?Db=gene&Cmd=ShowDetailView&TermToSearch=4076)*,* [*PAK6*](http://www.ncbi.nlm.nih.gov/sites/entrez?Db=gene&Cmd=ShowDetailView&TermToSearch=56924)*,* [*RB1*](http://www.ncbi.nlm.nih.gov/sites/entrez?Db=gene&Cmd=ShowDetailView&TermToSearch=5925) | [*ARID4B*](http://www.ncbi.nlm.nih.gov/sites/entrez?Db=gene&Cmd=ShowDetailView&TermToSearch=51742)*,* [*ARID4A*](http://www.ncbi.nlm.nih.gov/sites/entrez?Db=gene&Cmd=ShowDetailView&TermToSearch=5926)*,* [*JMJD3*](http://www.ncbi.nlm.nih.gov/sites/entrez?Db=gene&Cmd=ShowDetailView&TermToSearch=23135) | [*GRB10*](http://www.ncbi.nlm.nih.gov/sites/entrez?Db=gene&Cmd=ShowDetailView&TermToSearch=2887) | [*ACVR1*](http://www.ncbi.nlm.nih.gov/sites/entrez?Db=gene&Cmd=ShowDetailView&TermToSearch=90)*,* [*TSPAN9*](http://www.ncbi.nlm.nih.gov/sites/entrez?Db=gene&Cmd=ShowDetailView&TermToSearch=10867)*,* [*OSM*](http://www.ncbi.nlm.nih.gov/sites/entrez?Db=gene&Cmd=ShowDetailView&TermToSearch=5008)*,* [*FGFR2*](http://www.ncbi.nlm.nih.gov/sites/entrez?Db=gene&Cmd=ShowDetailView&TermToSearch=10818)*,* [*DICER1*](http://www.ncbi.nlm.nih.gov/sites/entrez?Db=gene&Cmd=ShowDetailView&TermToSearch=23405) | [*PIP5K3*](http://www.ncbi.nlm.nih.gov/sites/entrez?Db=gene&Cmd=ShowDetailView&TermToSearch=200576)*,* [*ARHGAP12*](http://www.ncbi.nlm.nih.gov/sites/entrez?Db=gene&Cmd=ShowDetailView&TermToSearch=94134)*,* [*JAKMIP1*](http://www.ncbi.nlm.nih.gov/sites/entrez?Db=gene&Cmd=ShowDetailView&TermToSearch=152789)*,* [*FZD6*](http://www.ncbi.nlm.nih.gov/sites/entrez?Db=gene&Cmd=ShowDetailView&TermToSearch=8323)*,* [*ARHGAP24*](http://www.ncbi.nlm.nih.gov/sites/entrez?Db=gene&Cmd=ShowDetailView&TermToSearch=83478)*,* [*ITPR1*](http://www.ncbi.nlm.nih.gov/sites/entrez?Db=gene&Cmd=ShowDetailView&TermToSearch=3708)*,* [*ARHGAP29*](http://www.ncbi.nlm.nih.gov/sites/entrez?Db=gene&Cmd=ShowDetailView&TermToSearch=9411)*,* [*MAP3K5*](http://www.ncbi.nlm.nih.gov/sites/entrez?Db=gene&Cmd=ShowDetailView&TermToSearch=4217)*,* [*RPS6KA5*](http://www.ncbi.nlm.nih.gov/sites/entrez?Db=gene&Cmd=ShowDetailView&TermToSearch=9252)*,* [*SMAD7*](http://www.ncbi.nlm.nih.gov/sites/entrez?Db=gene&Cmd=ShowDetailView&TermToSearch=4092)*,* [*MAP3K12*](http://www.ncbi.nlm.nih.gov/sites/entrez?Db=gene&Cmd=ShowDetailView&TermToSearch=7786)*,* [*FGF9*](http://www.ncbi.nlm.nih.gov/sites/entrez?Db=gene&Cmd=ShowDetailView&TermToSearch=2254)*,* [*RPS6KA3*](http://www.ncbi.nlm.nih.gov/sites/entrez?Db=gene&Cmd=ShowDetailView&TermToSearch=6197)*,* [*CSNK1G1*](http://www.ncbi.nlm.nih.gov/sites/entrez?Db=gene&Cmd=ShowDetailView&TermToSearch=53944)*,* [*ARHGAP1*](http://www.ncbi.nlm.nih.gov/sites/entrez?Db=gene&Cmd=ShowDetailView&TermToSearch=392)*,* [*MAP3K2*](http://www.ncbi.nlm.nih.gov/sites/entrez?Db=gene&Cmd=ShowDetailView&TermToSearch=10746)*,* [*ARHGEF11*](http://www.ncbi.nlm.nih.gov/sites/entrez?Db=gene&Cmd=ShowDetailView&TermToSearch=9826)*,* [*MAPK1*](http://www.ncbi.nlm.nih.gov/sites/entrez?Db=gene&Cmd=ShowDetailView&TermToSearch=5594)*,* [*SMAD4*](http://www.ncbi.nlm.nih.gov/sites/entrez?Db=gene&Cmd=ShowDetailView&TermToSearch=4089)*,* [*MAP3K8*](http://www.ncbi.nlm.nih.gov/sites/entrez?Db=gene&Cmd=ShowDetailView&TermToSearch=1326)*, LEFTY1, TGFBR2, IGF1* |
| **hsa-miR-145** | 3.25 | 8.469E-27 | *RASA2, CDC37L1, CCNL1, RASA1, CCNJ, CCND2, CDK4, MDM2* | *SMARCD1* | *PDCD4* | *SOX2, KLF4, MYC, POU5F1* | *PLCL2, PLCD4, RGS7, CAMK1D, JUNB, PTGFR, BMPR2, NLK, MAPK1, MAP4K4, MAP3K3, FZD7, BMPR2, ARHGAP26* |
| hsa-miR-23a | 3.21 | 4.683E-15 | *NEK6, MCM3AP, CDC40, CCND1, CDK5R1, CCNG1* | *SET8, JMJD2A, TGIF* | *CASP7* | *SOCS6, STAT5B* | *NIN, ARHGAP20, RRAS2, LRP5, RAB2, ARHGEF7, MAP3K12, NRK* |
| hsa-miR-498 | 3.20 | 4.730E-22 | [*TP53INP1*](http://www.ncbi.nlm.nih.gov/sites/entrez?Db=gene&Cmd=ShowDetailView&TermToSearch=94241)*,* [*CDK6*](http://www.ncbi.nlm.nih.gov/sites/entrez?Db=gene&Cmd=ShowDetailView&TermToSearch=1021)*,* [*CCNJL*](http://www.ncbi.nlm.nih.gov/sites/entrez?Db=gene&Cmd=ShowDetailView&TermToSearch=79616)*,* [*CCNJ*](http://www.ncbi.nlm.nih.gov/sites/entrez?Db=gene&Cmd=ShowDetailView&TermToSearch=54619) | [*JHDM1D*](http://www.ncbi.nlm.nih.gov/sites/entrez?Db=gene&Cmd=ShowDetailView&TermToSearch=80853)*,* [*SUV39H1*](http://www.ncbi.nlm.nih.gov/sites/entrez?Db=gene&Cmd=ShowDetailView&TermToSearch=6839) | [*MORF4L2*](http://www.ncbi.nlm.nih.gov/sites/entrez?Db=gene&Cmd=ShowDetailView&TermToSearch=9643) | [*DICER1*](http://www.ncbi.nlm.nih.gov/sites/entrez?Db=gene&Cmd=ShowDetailView&TermToSearch=23405)*,FGFR2,* [*SOCS3*](http://www.ncbi.nlm.nih.gov/sites/entrez?Db=gene&Cmd=ShowDetailView&TermToSearch=9021)*,* [*LIN28*](http://www.ncbi.nlm.nih.gov/sites/entrez?Db=gene&Cmd=ShowDetailView&TermToSearch=79727)*,* [*GABRB3*](http://www.ncbi.nlm.nih.gov/sites/entrez?Db=gene&Cmd=ShowDetailView&TermToSearch=2562)*,* | [*RAB28*](http://www.ncbi.nlm.nih.gov/sites/entrez?Db=gene&Cmd=ShowDetailView&TermToSearch=9364)*,* [*GABRA4*](http://www.ncbi.nlm.nih.gov/sites/entrez?Db=gene&Cmd=ShowDetailView&TermToSearch=2557)*,* [*RAB6A*](http://www.ncbi.nlm.nih.gov/sites/entrez?Db=gene&Cmd=ShowDetailView&TermToSearch=5870)*,* [*SMAD4*](http://www.ncbi.nlm.nih.gov/sites/entrez?Db=gene&Cmd=ShowDetailView&TermToSearch=4089)*,* [*RASIP1*](http://www.ncbi.nlm.nih.gov/sites/entrez?Db=gene&Cmd=ShowDetailView&TermToSearch=54922)*,* [*PIK3C2A*](http://www.ncbi.nlm.nih.gov/sites/entrez?Db=gene&Cmd=ShowDetailView&TermToSearch=5286) |
| hsa-miR-493* | 3.18 | 1.037E-38 | *-* | *-* | *-* | *-* | *-* |
| hsa-miR-487b | 3.11 | 5.027E-26 | *-* | *-* | *-* | *-* | [*LRP6*](http://www.ncbi.nlm.nih.gov/sites/entrez?Db=gene&Cmd=ShowDetailView&TermToSearch=4040)*,* [*IRS1*](http://www.ncbi.nlm.nih.gov/sites/entrez?Db=gene&Cmd=ShowDetailView&TermToSearch=3667) |
| hsa-miR-524-3p | 3.03 | 3.151E-37 | *-* | *-* | *PDCD10* | *-* | *-* |
| hsa-miR-143 | 3.01 | 1.656E-25 | *CNNM3* | *SMARCD2, DNMT3A* | *-* | *-* | *KRAS2, GLI3, RAB7, MAP3K7, ITPR1, DGKB, PTGS2, PRKCE* |

**Supplemental table 2.** Summary of miRNAs that are significantly up-regulated in DFs compared to iPSCs. The miRNAs highlighted in blue regulate the expression of pluripotency factors. For miRNAs target prediction and annotation to specific pathways, our own curation analysis and a combination of distinct software tools was used: TargetScan (http://www.targetscan.org/), miRanda (http://www.microrna.org/), Pictar (http://www.pictar.org/), Pubmed (http://www.ncbi.nlm.nih.gov/pubmed/). Analysis of miRNA seed sequences shows seven clusters that share very similar seed sequences and may indicate common transcriptional targets (**cluster 1**: hsa-miR-525-5p, hsa-miR-520a-5p; **cluster 2**: hsa-miR-519b-3p and hsa-miR-517*; **cluster 3**: hsa-miR-515-5p and hsa-miR-515-3p; **cluster 4**: hsa-miR-519b-3p and hsa-miR-517*; **cluster 5**: hsa-miR-517b and hsa-miR-517c; **cluster 6**: hsa-miR-518e, hsa-miR-518c, hsa-miR-518f, hsa-miR-518e*, hsa-miR-518b; **cluster 7**: hsa-miR-519d and hsa-miR-520h).
