# Supplementary material for: Brief Report: Inhibition of miR‐145 Enhances Reprogramming of Human Dermal Fibroblasts to Induced Pluripotent Stem Cells
Source: Stem Cells. 2015 Oct 9;34(1):246–51. doi: 10.1002/stem.2220 (PMC4982107; doi:10.1002/stem.2220)
Supplement: Supplementary file 5 — Supporting Information [file STEM-34-246-s005.docx]

**Supplemental Table 3**

| **Gene** | **Primer sequence** | **Universal ProbeLibrary number** |
| --- | --- | --- |
| *KLF4* | Left: gggagaagacactgcgtca | #52 |
|  | Right: ggaagcactgggggaagt |  |
| *C-MYC* | Left: gctgcttagacgctggattt | #66 |
|  | Right: taacgttgaggggcatcg |  |
| *GAPDH* | Left: agccacatcgctcagacac | #60 |
|  | Right: gcccaatacgaccaaatcc |  |
