# Supplementary material for: Brief Report: Inhibition of miR‐145 Enhances Reprogramming of Human Dermal Fibroblasts to Induced Pluripotent Stem Cells
Source: Stem Cells. 2015 Oct 9;34(1):246–51. doi: 10.1002/stem.2220 (PMC4982107; doi:10.1002/stem.2220)
Supplement: Supplementary file 6 — Supporting Information [file STEM-34-246-s006.docx]

**Supplemental material – Lako et al.**

**Materials and Methods**

Cell culture and miR-145 inhibition

Three different neonatal DFs (purchased form Lonza, Product code: CC-2509) were cultured in Knockout DMEM medium (Invitrogen, Life Technologies Ltd) containing 10% fetal bovine serum (PAA), 2 mM L-glutamine (Invitrogen, Life Technologies Ltd), 1x MEM non-essential amino acid solution, 1x Penicillin/Streptomycin (PAA) and β-mercaptoethanol (Sigma-Aldrich). DFs were transduced using lentiviral particles containing hsa-miR-145-5p inhibitor (Genecopoeia) at MOI = 40 in the presence of 5 μg/ml Polybrene (Sigma-Aldrich). Transduced cells were selected for Hygromycin resistance (50-75 μg/ml). For transient miR-145 inhibition, 1 x 10^5^ DFs were transfected with 100 pmoles miR-145 *mir*Vana® miRNA inhibitor (Life Technologies Ltd) using Neon transfection system (Invitrogen). Transfection was carried out by two 1600 V pulses for 20 ms.

Reprogramming to iPSCs

Cells were transduced using CytoTune®-iPS Sendai Reprogramming Kit (Product number A1378001) (Life Technologies Ltd) according to manufacturer’s instructions. The efficiency of iPSCs generation was assessed by alkaline phosphatase (AP) activity staining using Alkaline Phosphatase Blue Substrate (Sigma-Aldrich) and by TRA-1-60 expression, as determined indirect immunofluorescence. Cells were washed with PBS, fixed by 4% paraformaldehyde for 10 minutes at room temperature, washed again with PBS, and incubated overnight at 4°C with primary antibody against TRA-1-60 (MAB4360, Merck Millipore). Then cells were washed three times with PBS and incubated with Alexa 488-conjugated secondary antibody and observed under fluorescent microscope.

Differential miRNA expression analysis

DFs were grown in standard culture conditions, as described above. iPSCs were cultured in undifferentiated, pluripotent state as discrete colonies on feeder layers of mitotically inactivated mouse embryonic fibroblasts (MEF) with medium containing Knockout Dulbecco’s modified Eagle’s medium (DMEM) (Invitrogen, Paisley, U.K., http:// www.invitrogen.com), 1 mM L-glutamine (Invitrogen), 100 mM nonessential amino acids (Invitrogen), 20% serum replacement (Invitrogen) and 8 ng/ml fibroblast growth factor 2 (FGF2; Invitrogen). To avoid feeder cell contamination, two passages before RNA extraction cells were transferred to Matrigel Basement Membrane Matrix (BD Biosciences) coated dishes and cultured in MEF-conditioned media. 100ng of total RNA from one cell line of DFs and iPSCs, derived from the same DF cell line, was labelled and hybridised to the Agilent human miRNA (V3) 8X15K microarray (total number of detected miRNAs on platform: 961) according to manufacturer’s instructions. In brief, the expression data was normalised using quantile normalisation [1] and differential miRNA expression was estimated using Limma package [2] from Bioconductor. Statistical significance (adjusted p-values) according to Benjamini-Hochberg [3] was used to select the miRNAs that were differentially expressed between DFs (passage 5) and iPSCs (passage 20). The microarray data were confirmed using RT-qPCR on 14 different miRNAs (data not shown). The microarray data are available in the Gene Expression Omnibus (GEO) database (http://www.ncbi.nlm.nih.gov/gds/) under the accession number GSE68672.

miRNA quantification

miRNA isolation, transcription to cDNA, and quantification was performed using TaqMan microRNA Cells-to-Ct kit (4391848, Life Technologies Ltd) according to manufacturer’s instructions. Briefly, cells were washed in PBS, lysed in Lysis Buffer containing DNase I, and incubated at room temperature for 8 minutes. To inhibit the lysis reagents, Stop Solution was added to lysate and was incubated at room temperature for 2 minutes. Cell lysates were then reverse transcribed in reactions containing RT primer for the target of interest supplied with a TaqMan MicroRNA Assay (44279753) (for RNU6B assay ID number: 001093, for miR-145 assay ID number: 002278, for let-7b assay ID number: 002619, for miR-143 assay ID number: 002249, for miR-363 assay ID number: 001271, for miR-18a assay ID number: 002422, for miR-367 assay ID number: 000555), Life Technologies Ltd). RT product was amplified by real-time PCR (Applied Biosystems 7900 or Roche LightCycler® 480 PCR instruments) using TaqMan Universal PCR Master Mix with probes included in TaqMan MicroRNA Assay. The relative expression was calculated by normalisation to *RNU6B* expression.

Western blot analysis

Western blot analysis was performed as described in [4]. Primary antibodies were used as follows: Vimentin (#5741), N-Cadherin (#13116), ZO-1 (#8193), Snail (#3879), E-Cadherin (#3195), Klf4 (#4038), c-Myc (#5605) all purchased from Cell Signaling Technology. β-actin (AB1801) (Abcam), Sox2 (AB5603) (Millipore), and Mdm2 (sc-56154), Wip1 (sc-20712), Oct3/4 (sc-5279), CDK4 (sc-260) purchased from Santa Cruz Biotechnology.

Flow cytometry

Cells were harvested using trypsin-EDTA (0.05%) for 5 minutes. Flow cytometry was performed using Cytomics FC500 (Beckman Coulter, CA, USA). Data were analysed using FlowJo software ([www.flowjo.com](http://www.flowjo.com)). At least 10,000 events were analysed for each experiment.

Wound Healing Assay

Cells were scratched by a fine pipette tip, then culture medium was changed. The cell migration distance was determined by measure the wound width. The relative recovery rate was calculated as: (initial wound width - wound width at the time of measurement)/ initial wound width x100.

Real-time qPCR

RNA was isolated using RNeasy Mini Kit (Qiagen) according to manufacturer’s instructions. RNA was transcribed to cDNA using Transcriptor First Strand cDNA Synthesis Kit (Roche). Real-time qPCR was performed using FAM-labelled probes from Universal ProbeLibrary (Roche). The sequences of primers and probes are shown in Supplemental Table 3. Real-time qPCR was performed using Roche LightCycler® 480 PCR instrument. The relative expression was calculated by normalisation to *GAPDH* expression.

MiRNA target prediction

For miRNAs target prediction and annotation to specific pathways, our own curation analysis and a combination of distinct software tools was used: TargetScan (http://www.targetscan.org/), miRanda (http://www.microrna.org/), Pictar (http://www.pictar.org/), Pubmed (<http://www.ncbi.nlm.nih.gov/pubmed/>).

Statistical analysis

Statistical analysis was performed using MS Office Excel (Microsoft). Statistical significance was determined by paired two-tailed t-test at levels of P < 0.05 (*), P < 0.01 (**), and P < 0.001 (***).

**Supplemental Materials References**

1. Bolstad BM, Irizarry RA, Astrand M, et al. A comparison of normalization methods for high density oligonucleotide array data based on variance and bias. **BIOINFORMA. OXF. ENGL.** 2003;19(2):185–193.

2. Smyth GK. Linear models and empirical bayes methods for assessing differential expression in microarray experiments. **STAT. APPL. GENET. MOL. BIOL.** 2004;3:Article3.

3. Benjamini Y, Hochberg Y. Controlling the False Discovery Rate: A Practical and Powerful Approach to Multiple Testing. **J. R. STAT. SOC. SER. B METHODOL.** 1995;57(1):289–300.

4. Bárta T, Vinarský V, Holubcová Z, et al. Human embryonic stem cells are capable of executing G1/S checkpoint activation. **STEM CELLS DAYT. OHIO** 2010;28(7):1143–1152.

Figure Legends

Figure S1: Wound healing assay upon miR-145 inhibition. Bars represent relative recovery rate at specific time-points upon wound scratching, graph shows data from experiment using two independent cell lines. Error bars represent standard errors.

Figure S2: Expression of miR-145 in DFs 24 hours upon its transient transfection with miR-145, as determined by RT-qPCR. Error bars show standard deviation (n=6). Stars show statistical significance [P < 0.05 (*), P < 0.01 (**), and P < 0.001 (***)].

Figure S3: Expression of C-MYC and KLF4 at 24, 48, and 72 hours upon transient miR-145 inhibition, as demonstrated by RT-qPCR. Error bars show standard deviation (n=3). Stars show statistical significance [P < 0.05 (*), P < 0.01 (**), and P < 0.001 (***)].
